# Toward Precision Psychiatry: Statistical Platform for the Personalized Characterization of Natural Behaviors

**DOI:** 10.3389/fneur.2016.00008

**Published:** 2016-02-02

**Authors:** Elizabeth B. Torres, Robert W. Isenhower, Jillian Nguyen, Caroline Whyatt, John I. Nurnberger, Jorge V. Jose, Steven M. Silverstein, Thomas V. Papathomas, Jacob Sage, Jonathan Cole

**Affiliations:** ^1^Psychology Department, Rutgers University, New Brunswick, NJ, USA; ^2^Rutgers Center for Cognitive Science, Rutgers University, New Brunswick, NJ, USA; ^3^Computer Science Department, Center for Biomedical Imaging and Modeling, Rutgers University, New Brunswick, NJ, USA; ^4^Department of Psychiatry, Institute of Psychiatric Research, Indiana University School of Medicine, Indianapolis, IN, USA; ^5^Department of Physics, Indiana University, Bloomington, IN, USA; ^6^Department of Cellular and Integrative Physiology, Indiana University, Indianapolis, IN, USA; ^7^Department of Psychiatry, Rutgers University Robert Wood Johnson Medical School, New Brunswick, NJ, USA; ^8^Department of Biomedical Engineering, Rutgers University, New Brunswick, NJ, USA; ^9^Movement Disorders, Rutgers University Robert Wood Johnson Medical School, New Brunswick, NJ, USA; ^10^Poole Hospital and Bournemouth University, Poole, UK

**Keywords:** precision phenotyping, sensory–motor noise, autism spectrum disorders, Parkinson’s disease, schizophrenia, deafferentation

## Abstract

There is a critical need for new analytics to personalize behavioral data analysis across different fields, including kinesiology, sports science, and behavioral neuroscience. Specifically, to better translate and integrate basic research into patient care, we need to radically transform the methods by which we describe and interpret movement data. Here, we show that hidden in the “noise,” smoothed out by averaging movement kinematics data, lies a wealth of information that selectively differentiates neurological and mental disorders such as Parkinson’s disease, deafferentation, autism spectrum disorders, and schizophrenia from typically developing and typically aging controls. In this report, we quantify the continuous forward-and-back pointing movements of participants from a large heterogeneous cohort comprising typical and pathological cases. We empirically estimate the statistical parameters of the probability distributions for each individual in the cohort and report the parameter ranges for each clinical group after characterization of healthy developing and aging groups. We coin this newly proposed platform for individualized behavioral analyses “*precision phenotyping*” to distinguish it from the type of observational–behavioral phenotyping prevalent in clinical studies or from the “one-size-fits-all” model in basic movement science. We further propose the use of this platform as a unifying statistical framework to characterize brain disorders of known etiology in relation to idiopathic neurological disorders with similar phenotypic manifestations.

## Introduction

**Figure 1:**
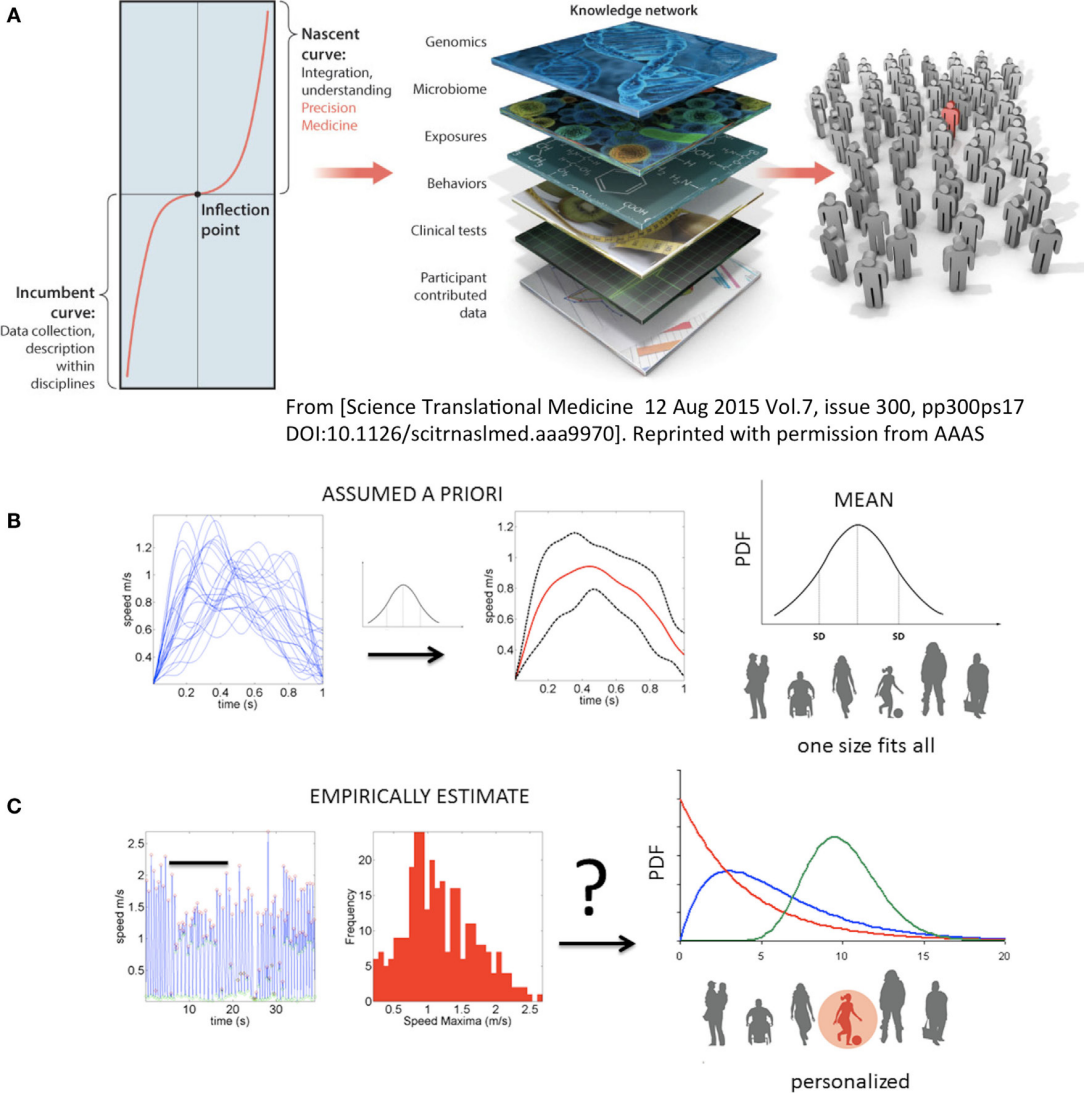
**Toward true personalized medicine in behavioral sciences**. **(A)** “An inflection point marks an opportunity or moment of dramatic change between the first, or incumbent curve, marking steady progress, and a second, or nascent, curve, indicating transformation and accelerated progress. In biomedical research, health, and health care, we are at an inflection point, poised for precision medicine,” quoted from Hawgood et al. (1). **(B)** In the behavioral and mental health sciences personalized medicine needed to achieve the inflection point leading to accelerated transformation is not yet possible. Traditional statistics in use today prevent the development of Precision Psychiatry and call for a disruptive methodology that changes the course of current basic research and patient care in the mental health and behavioral disciplines. Example shows the current “state-of-the-art” approach to motion analyses in behavioral sciences. Under this approach, researchers may take a handful of trials and average a certain parameter (e.g., the speed values) under assumption of normality. Critically, subtle fluctuations in behavioral performance are smoothed out as noise. That average behavior is used as a model to compare performance of individual participants. Note that the assumed theoretical Gaussian distribution leads to a one-size-fits-all treatment of behavioral data, making this statistical approach incompatible with key tenets of precision medicine. **(C)** New statistical platform for individualized behavioral analyses. Continuous behavioral markers (e.g., physiological motion signals) naturally show fluctuations in performance (e.g., amplitude and timing) that accumulate information toward an expected value, then shift signatures in non-stationary fashion [bar indicates snapshot of behavior in **(B)** along the continuous stream]. The probability distribution function (PDF) is continuously empirically estimated. A given individual is rather characterized by a family of PDFs with individualized rate of accumulation and change of these stochastic parameters as a function of treatment and disease progression.

Precision medicine is a new approach to acquire and integrate knowledge from biomedical research and clinical practice (1). It is a computation-enabled platform poised to radically transform the ways in which we currently conduct biomedical research and patient care by integrating personal information across many layers, from genes to behavior (Figure 1A). The personalized approach has been successful in areas such as cancer research and treatment. In contrast, the disciplines of mental health and social sciences tend to follow a “one-size-fits-all” approach and rely primarily on the bottom layers of the knowledge network – self-reports and clinical ratings and their interpretation – but not as much on objective physical measurements tailored to the individual.

In recent years, the need to shift from symptom- and interpretation-based approaches in neurological disorders and mental illnesses to more objective methods has been voiced in a various ways. One such method is the Precision Psychiatry initiative of the National Institute of Mental Health (NIMH), where various task forces have been created to achieve more objective science that unveils biological signatures of core *dimensions* of functioning (e.g., cognition, positive valence systems, and arousal), as they are expressed across neuropsychological and neurological disorders on a spectrum. More explicitly, the Research Domain Criteria (RDoC) initiative (2) is one of the various attempts to bring basic research on mental illness to a new level of rigor that also helps accelerate progress. Surprisingly, however, the current RDoC matrix does not include a dimension of sensory–motor function (3). Here, we argue that movement and its sensation could be a great ally in tailoring research and treatment to the patient’s needs and inherent predispositions. As such, it may be a good idea to include sensory–motor function as a dimension within RDoC and to incorporate objective and movement-based outcomes into research on brain disorders. For the remainder of this paper, we demonstrate a novel quantitative method that we believe is particularly useful in this regard.

A simple experimental paradigm is presented, with a new statistical method for individualized behavioral analyses and a new kinematic data type (explained in Section “New Data Type and Different Assessment of Motor Variability”). Both form the basis of a unifying platform to help personalize research and patient care within the field of disorders of the central and peripheral nervous systems. More specifically, we address data analyses differently from traditional approaches (Figure 1B) and use the new platform (Figure 1C) to empirically estimate the individualized stochastic signatures of the moment-by-moment fluctuations in performance across several clinical and nonclinical populations. These populations range from typical controls of various age groups (children, young college students, middle-aged, and elderly participants) to patients of various types. The latter include Parkinson’s disease (PD) at mild and severe stages, schizophrenia (SZ) patients of different ages, and individuals with a diagnosis of autism spectrum disorder (ASD), from various age groups. In addition, we include parents of individuals affected by ASD to investigate whether their motor patterns fall within the signatures uncovered in the normal control groups. Finally, we describe the data from a patient who lost peripheral sensory inputs from touch, pressure, and movements, resulting in loss of proprioception from the neck down, but whose motor nerves are unaffected. We provide an overview of the statistical parameters that are empirically estimated from the movement kinematics of all participants and demonstrate that these reveal fundamentally different features across disorders, as well as overlapping features. Results are discussed in the context of Precision Medicine and Precision Psychiatry. In particular, we emphasize that these analytic methods produce fine-grained variables that are well-suited to bridging the gap between coarse behavioral descriptors and genetic factors, which may underlie some sensory–motor noise signatures across disorders.

## Methods

### Subjects

To empirically estimate the ranges of statistical parameters underlying kinematics data, we use data from various subject groups. All subjects provided written informed consent on forms approved by The Rutgers University Institutional Review Board (IRB) or Indiana University IRB. All protocols were approved by the IRB committees, in compliance with the Helsinki Act. Clinical records were obtained in compliance with the Health Insurance Portability and Accountability Act (HIPAA). Parents of the subjects with ASD signed the IRB approved consent on behalf of their child/adult participant with ASD. Table S1 in Supplementary Material summarizes the demographic characteristics of the 176 participants.

The control subjects were subdivided into four broad groups including young children (CT1), young college students (CT2), middle-aged subjects (CT3) and the elderly subjects. In line with previous research indicating maturity of pointing kinematics after 4 years of age (4), we further subdivided the CT1 group. Within CT1, we examined individuals between 3 and 4 years of age (CT1a) and those between 5 and 10 years of age (CT1b). Another control group was the parents of a subset of the children affected by ASD. The latter control group had no ASD diagnosis but their movements were visibly different from those of other control middle-aged individuals. This prompted us to perform this comparison, despite a lack of clinical diagnosis. From a subset of the parents, we estimated that the age of the cohort ranged between 32 and 39 for mothers and 32 and 44 for fathers.

The demographic information and clinical scores for the ASD group are shown in Table S2 in Supplementary Material. Within this group, ASD1 was composed of 3–12-year-old participant and ASD2 was comprised of 13–25-year-old participants.

A PD cohort was also included. They were recruited from the PD support group of the Rutgers-Robert Wood Johnson Medical School’s Movement Disorders Center. The PD group was subdivided into 9 subjects with mild-to-moderate disease severity (PD1) who were ambulatory, independent, and with some visible resting tremor but without visible action tremor at the time of the visit, and 17 subjects (severe PD2) who had very impaired mobility, some of whom were ataxic and some with freezing of gait. The latter group generally needed assistance to walk and had visible action and resting tremor. Table S3 in Supplementary Material shows the demographics of both groups.

A group of eight elderly individuals (ages 75–77 years old) with no formal diagnosis of a movement disorder was included as a control group for the PD groups comprising a broad range of ages (46–77 years old). These subjects were part of an earlier study (4) where we had aimed to statistically characterize action tremor during pointing behavior in typically aging individuals.

A group of 23 patients with SZ was included in the study to ascertain their motor signatures in relation to the other cohorts and to age- and sex-matched controls. SZ patients were recruited from Rutgers University Behavioral Health Care clinics. Patients were either enrolled in a daily partial hospital program (PHP) or were outpatients who only required biweekly or monthly visits to health-care providers. There were 10 patients enrolled in the PHP and 13 patients in the outpatient program. Table S4 in Supplementary Material reports demographics and Table S5 in Supplementary Material reports the Frontal System Behavior Scale (FrSBE) self-rating scores for executive dysfunction.

Finally, a special subject (IW) without proprioception, secondary to deafferentation (5) was included. This subject suffered a lack of proprioception and touch from the C3 level down due to acute sensory neuronopathy syndrome. This syndrome led to irreversible sensory nerve destruction at the dorsal root ganglia level of fibers conducting touch, pressure, and movement information from the periphery to the central control centers of the brain. The motor nerves and the movement output of the deafferented subject were unaffected. This particular participant has learned to move in a controlled manner again using mental concentration on movements with visual supervision to help close the feedback loops. The signal that we are capturing in most subjects contains a blend of motor and sensory noise. This participant provides an example of motor noise in the absence of sensory afferents conducting movement information. We use the locations of his estimated signatures of speed profile-dependent variability during visual feedback and in the dark. These points located on the Gamma parameter plane (see Methods below) serve as a reference to anchor the data from other patients.

Due to the individualized nature of this approach, whereby each participant is their own control [i.e., examined with respect to its own empirically estimated family of probability distribution functions (PDFs) from the motion parameters], it is not necessary to match the number of participants in each clinical category to the exact number of controls of a given age/sex. However, the controls included a broad range of ages from both sexes, spanning the age and sex range of the participants with a clinical diagnosis. In this sense, the aim of the age- or disease-stage subdivisions was to use the new methods to show how to obtain the statistical summaries for the kinematic parameters of interest for representative cross-sections of the typical population. However, we emphasize that the individualized scatterplots that we provide can also be examined blindly (i.e., without *a priori* imposed clinical labels).

### Experimental Setup

**Figure 2:**
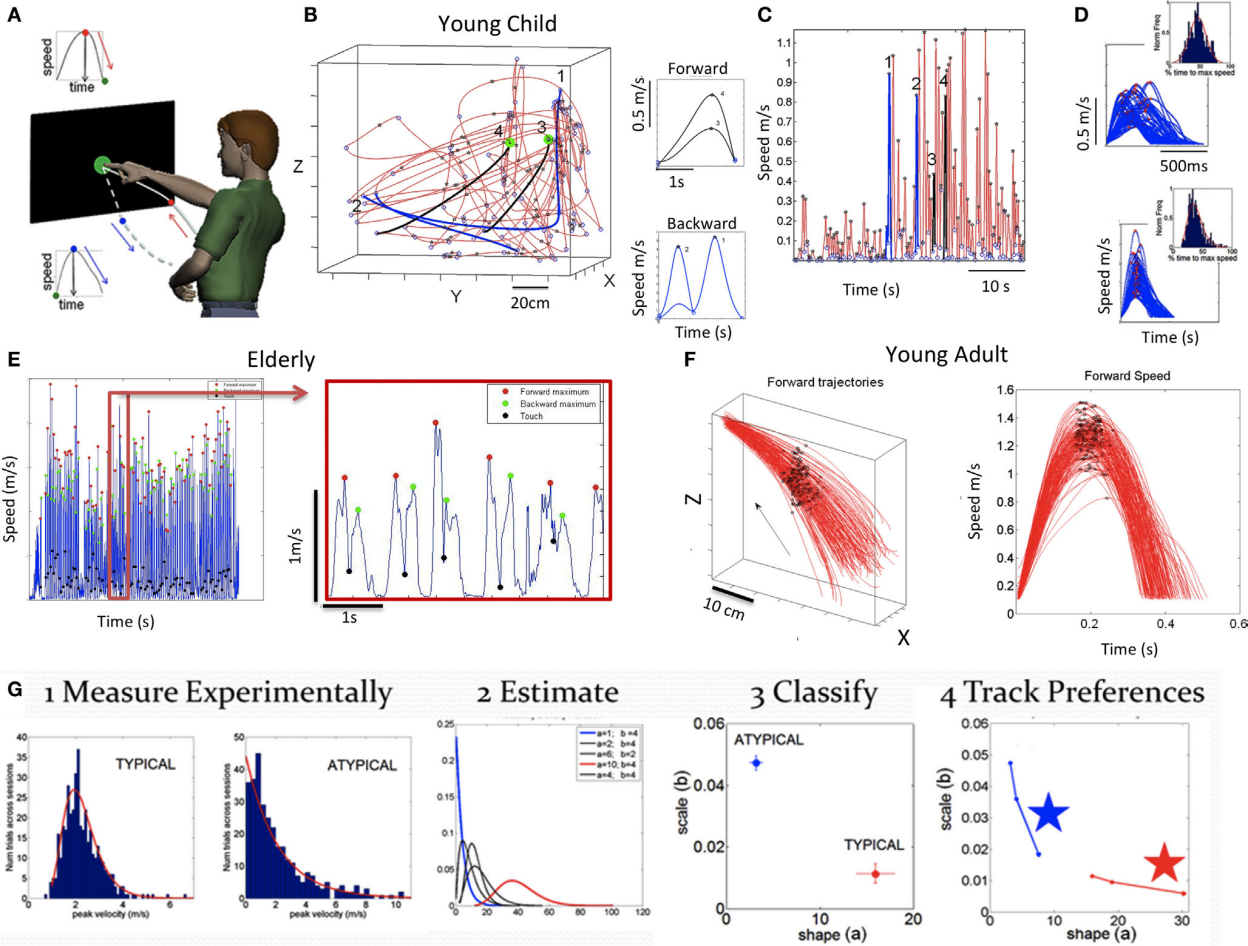
**Basic experimental paradigm, sample raw data and methods**. **(A)** Full forward and back pointing loops continuously measured as they unfold in a forward segment deliberately intended toward the target and a spontaneous (uninstructed) retraction away from the target. Touch screen is used to automatically register the end of the goal directed motion (when the hand speed is at near zero velocity and its position is at near zero-distance to target). At this point, the target-hand distance and the speed increase as the hand starts retracting away from the target. This position-speed hand configuration marks the beginning of retraction movement segments. The ending of those segments are determined by near zero-velocity criteria marking pauses in the continuous motions. **(B)** Trajectories and corresponding speed profiles from continuous motions of a young child naturally performing the task without constraints. Data extraction relies as previously described on hand positional distance and speed criteria. Sample trajectories for two sample forward (black) and backwards (blue) movement trajectories that were automatically detected using these criteria are shown. Notice that end point errors can be large in young children, particularly those in the spectrum. Therefore, no restrictions are imposed on target accuracy. The focus is rather on the spread of the moment-by-moment peak velocity (i.e., fluctuations in performance) during a full pointing loop. **(C)** Discrete segments of speed profiles from the 3D hand trajectories are color coded to identify the ones marked on the 3D plot in **(B)** and on the continuous speed plot in **(C)** (black forward, blue backwards). Numbers mark these segments, aligned at movement onset. Landmarks of the continuous motions are the peak speeds (stars) between local minima (circles). **(D)** Examples of speed profiles from retractions aligned to movement onset in a child with ASD (top) and a CT1 child (bottom) with inset histograms showing the corresponding normalized frequency of the time to reach the local maxima. **(E)** Sample speed profiles continuously taken from a representative elderly participant with landmarks (speed minima, speed maxima, and screen touches) zoomed in for clarity. **(F)** Hand trajectories forward to the target from a young representative control (CT2 group) and corresponding speed profiles aligned to movement onset. Black dots mark the spread of the speed maxima along the forward trajectories and their temporal speed profile from trial to trial (for clarity, only forward segments are shown aligned to movement onset but backwards are similar in the opposite direction). **(G)** Analytical steps to empirically estimate the statistical signatures of these motions (see text for detailed explanation).

Figure 2A depicts the basic experimental paradigm consisting of a full pointing motion forward to the target and back to rest. The forward segment toward the target is instructed and goal directed. As such, it results in a deliberate pointing action. In contrast, the retraction away from the target, after the pointing action ended, is spontaneously performed without instructions. Pointing accuracy was not required, as the continuously periodically alternating nature of this motor action was more relevant to our analyses than the accuracy of the pointing act. The experiment took place under conditions of visual feedback.

In the case of the deafferented subject, we studied the pointing movements across several conditions. These included pointing in complete darkness while relying only on the memory of the target (flashed for a second); pointing in the dark with a light-emitting-diode (LED) attached to the moving finger but no visual feedback from the target; and pointing in the dark with no LED on the finger but with continuous visual guidance from the target ON throughout the motion. We separated this subject’s performance according to visual feedback conditions: dark vs. vision.

### Instructions to the Participants

Participants sat in a chair facing the target location at a comfortable distance for reaching (i.e., they did not have to completely stretch the arm; see schematics in Figure 2A). They were instructed to touch the target when it was presented. The forward motions toward the target were explicitly instructed with the words “Touch the target when it appears.” In marked contrast, the retracting motions from the target back to rest were not instructed – participants spontaneously performed these movements. In this sense, we underscore that the retracting motions were automatically performed without any explicit visual target. Our previous work had demonstrated striking differences between the kinematics of the instructed forward motions and the spontaneous retractions. These differences manifest in families of reaching motions such as pointing (6–8), reach-to-grasp (9), and also in martial arts routines requiring forward and back motions (10, 11). Building on these previous results, we examined the spatiotemporal features of these two separate movement types in order to assess how such differences may manifest across neuropsychiatric and neurological disorders.

### New Data Type and Different Assessment of Motor Variability

Since Bernstein’s work on the importance of motor variability (12) to central control of self-produced movements, many studies have assessed the variability of kinematic parameters. In the reaching domain, these have included end point error (13, 14), speed (15), and joint angles (16, 17), among many others. In all cases, the noise-to-signal balance has been examined under the assumption of normality. Variability is thus described relative to a central value (the assumed mean). Often, only a small number of trials are used to determine the fluctuations of a given parameter around that mean. To this end, the average of that parameter is obtained, and the ±deviations around the central value are computed assuming the symmetric (theoretical) Gaussian PDF. This is illustrated for the case of the speed profile taken as a set of movement parameters in Figure 1B. The assumptions of Gaussian PDF extend to stochastic models of motor control (13, 18) and Bayesian estimation-based models (19). To the best of our knowledge, the PDFs most likely underlying kinematic parameters of hand movements across disorders of the nervous systems have not been empirically estimated. Furthermore, estimations of such PDF in cross-sections of the normal population as a function of age groups have not been performed either. Such estimations are necessary to assess the noise-to-signal ratios of movement parameters across the general population. This is in contrast to assuming a theoretical PDF *a priori* to describe the normal subset of the population without empirically estimating it. Specifically, we do not know how sensory–motor priors develop under normal or atypical conditions, how they may shift with typical aging, or how they may change with a degenerative disorder of the nervous system.

We assess the continuous time series of movement speed to empirically estimate the noise-to-signal ratios of velocity-dependent parameters from hand movement trajectories. The raw data in this case are the speed profile (such as that depicted in Figure 1C) continuously tracked by motion capture sensors. The waveform of the speed temporal profile from point-to-point varies, as it depends on the curvature of the underlying positional trajectory, tied as well to internal parameters such as the rotation of joints and the changes in muscle states (20, 21). Yet, regardless of the shape of the hand path, the speed rises during the acceleration phase to reach a maximum value and then generally decays in the deceleration phase to a stop or pause en route to the target. In the motor control literature such submovements and their variability around the assumed mean are commonly studied [e.g., Ref. (22)]. For highly automated straight point-to-point hand movements, their shape is approximately symmetric (23, 24).

Our interest, however, is not in the variations of the hand’s submovements around a mean value obtained under the assumption of a theoretical symmetric (e.g., Gaussian) distribution. Instead, we are interested in the accumulation (and the rate of change) of minute fluctuations in performance that occur from moment to moment in the parameters associated with the speed of motion. These include (among others) the fluctuations in maxima and those in the time to reach the velocity peak from the last pause or stop instance. As the motion of the arm-hand linkage is repeated, these minute fluctuations in speed accumulate and give rise to various frequency distributions. The shape and scale (dispersion – see Step 3 below) of these distributions can be estimated with high confidence to empirically approximate, along a continuum, the family of PDFs most likely describing the underlying random process. Once again, this is in contrast to assuming a theoretical PDF *a priori*.

To experimentally measure fluctuations in the speed amplitude from moment to moment, we accumulate the changes in the speed maximum [termed here peak velocity (PV)]. Since the speed waveform localized between two minima within the time series of speed (e.g., Figure 1C) may change the shape and amplitude from local speed minima to local speed minima, we must first normalize it (25). To this end, we obtain for each minima-to-minima segment the following index:

$$\text{nPVindex}=\frac{\text{PV}}{\text{PV}+\text{Average}(V_{\text{min to min}})}$$

Here, PV denotes the peak velocity (speed maximum) and the denominator contains the sum of the PV and the average speed between two consecutive local speed minima. We term this the normalized PV index. This normalization process also avoids possible allometry effects due to differences in the sizes of the limbs of the subjects (e.g., children vs. adults) (26). Larger values of this index indicate slower movements on average, since smaller averaged speed values in the denominator result in higher values of the index. These would be expected in the PD population that suffers from bradykinesia but not in the typical controls (for example).

The fluctuations in the overall profile, as determined by the changes in amplitude and timing of each peak, provide information about the individual rate of change of these variables as the nervous system of the person generates and then experiences them. Examples of the accumulation of variations in amplitude and timing in the time series of speed profiles are depicted in Figure 1C. We emphasize that this treatment of the variability problem fundamentally differs from traditional approaches, whereby it is assumed that the speed parameters follow a Gaussian distribution with known mean and variance. Therefore, further statistical analyses typically involve testing shifts in the mean/variance above chance and the use of parametric models assuming population statistics under a “one-size-fits-all” approach (Figure 1B).

### Analytical Techniques

In a series of papers, we have described these statistical techniques [e.g., Ref. (8, 11)]. A brief summary for the purposes of this report has four main stages, as detailed in Figure 2G:

(*Step 1*) Acquire time-series data (e.g., kinematics) from continuous trajectories of unconstrained target-directed pointing movements in three dimensions. Figure 2B shows sample data from the naturalistic hand trajectories of a young child. Figure 2C shows temporal speed profiles and the main landmarks used to study some of the patterns of velocity-dependent variability. These include the velocity peaks (meter per second) and the time (milliseconds) to reach those peaks from the local minima, among others. Sample speed profiles automatically extracted from the continuous data are also shown in Figure 2D for ASD and CT1 children of comparable age. Sample data from adults are shown in Figure 2E (elderly participants) and Figure 2F (young CT2).
(*Step 2*) Plot the frequency histograms (Figure 2G step 1) of the parameter of interest (e.g., the normalized PV index) using optimal binning (27, 28) and estimate the underlying family of probability distributions of speed profile-dependent parameter that best characterizes the trial-to-trial fluctuations in performance for each individual (Figure 2G step 2). Besides individual estimation, this procedure can also be done for cohorts of participants with a neurological disorder or typically developing individuals.
(*Step 3*) Use maximum likelihood estimation empirically to obtain – from the data – the values and ranges of the shape (*a*) and scale (*b*) parameters of the continuous Gamma family of probability distributions. The Gamma PD F is given by:
  $$y=f(x\text{ | }a,b)=\frac{1}{b^{\text{a}}\Gamma (a)}x^{\text{a}-1}e^{\frac{-\text{x}}{\text{b}}}$$
  in which *a* is the shape parameter, *b* is the scale parameter, and γ is the Gamma function (29). We then plot the estimated Gamma parameters for each participant with 95% confidence intervals on the (*a*, *b*)-Gamma parameter plane. Using this method, we localize the individual participant and can compare each subject’s location to those of the other subjects (Figure 2G step 3). Here, we also look at the overall data to identify self-emerging clusters and patterns, particularly in relation to a participant with a disorder of known etiology. For example, one could use this methodology to identify clusters or patterns across participants with a disorder that is clinically diagnosed based on symptoms alone vs. participants with the same clinical disorder, but with known genetic origins. By color coding the scatterplot points based on clinical criteria, we may be able to help with interpretation. In such cases clusters of participants with idiopathic diagnosis and similar symptoms may be studied in relation to those whose symptoms are of known etiology.
  The noise-to-signal ratio [i.e., the Fano Factor (30), FF = empirically estimated Gamma variance/empirically estimated Gamma mean] is also obtained. The Gamma mean is given by μ = *a* × *b* and the Gamma variance is given by σ = *a* × *b*^2^. Notice that the noise-to-signal ratio, the Fano Factor is also in this case the Gamma scale parameter $b=\frac{\sigma^{2}}{\mu }=\frac{a\times b^{2}}{a\times b}$ (29). This is important as we are assessing the levels of noise in relation to the empirical estimation of the Gamma parameters from the data as a function of clinical group type. Higher levels of noise correspond to an increase in the *b scale* parameter along the vertical axis of the Gamma plane, whereas lower levels of noise correspond to lower values of the scale parameter.
  It is also important to emphasize that when the *shape* parameter *a* of the Gamma family is equal to 1 (*a* = *1*), the data follow the memoryless exponential probability distribution, a special case in the Gamma family. This is the most random distribution, coined as “memoryless” because events in the past do not accumulate information predictive of events in the future (29). Larger values toward the right of the shape axis on the Gamma (*a*, *b*)-plane tend toward the symmetric distributions, with a various skewed distributions in between the two extremes (31).
  In the text, we will refer to the level of randomness by examining the value of the empirically estimated shape parameter. When close to *a* = 1, the shape denotes the memoryless Exponential distribution. When increasing the shape value to the right of the horizontal axis, we will refer to the accumulation of information toward the prediction of an expected value, away from *a* = 1, toward the Gaussian range of the Gamma plane. Likewise, we will refer to higher or lower noise levels according to the empirically estimated *b* Gamma-scale parameter value, which is the FF.
(*Step 4*) Repeat this estimation procedure to characterize the rate of change of the Gamma parameters’ stochastic trajectory. This step can detect conditions and stimuli that accelerate the change in the parameters down and to the right (i.e., to the right along the shape axis) away from random regimes of the Gamma plane (i.e., when *a* = *1*) and down along the scale axis, away from high noise-to-signal ratio values. Figure 2G step 4 marked with stars indicates the largest step, which illustrates a stochastic trajectory that is moving in the abovementioned statistically desirable direction. Results featured in this panel demonstrate this evolution within an ASD vs. CT experimental intervention (32). By examining large incremental steps in the stochastic signatures that lower the noise and increase the shape value toward Gaussian models, we can infer a range of implications, including which context is most appropriate to improve motor output within sensory manipulations? What effect a psychotropic medication dosage may have on each person? or Which therapeutic exercise, among a set of routines, is the most beneficial to the persons’ statistical motor patterns?

## Results

Figure 4 provides a color-coded map of the summary statistics of all participants. Figure 5 summarizes the *p*-values from pairwise statistical comparisons in matrix form across all neuropsychiatric/neurological disorders within the study. Tables S6–S9 in Supplementary Material summarize the statistical results of this study. Each supplementary table is accompanied by a Figures S3–S6 in Supplementary Material that helps visualize the results for each patient subgroup. Moreover, Figures S1 and S2 in Supplementary Material examine ensemble temporal kinematics data as per clinical diagnosis in relation to healthy controls. Below we discuss each finding separately.

### Typical Controls May Shift Statistical Signatures across the Life Span

**Figure 3:**
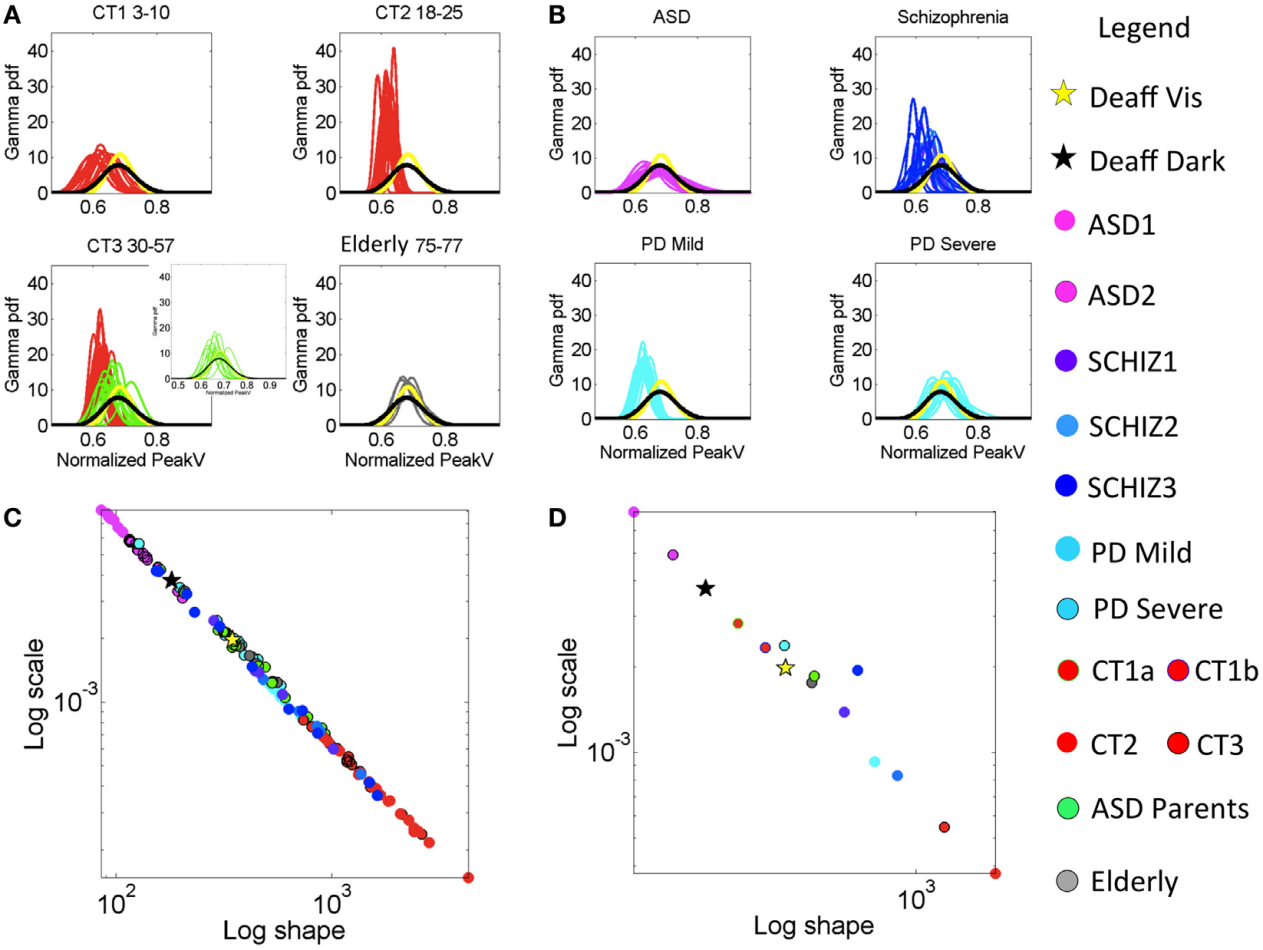
**Characterization of the stochastic signatures of the normalized peak velocity index estimated from fluctuations in this parameter across individualized task performance as well as for the group patients under consideration**. **(A)** Estimated PDFs for control groups. Top-left panel is the group of typically developing (control) children CT1 between 3 and 10 years old. Top-right panel CT2 contains the young participants (18–15 years old). Bottom-left panel CT3 is from 30- to 57-year-old participants. This panel also contains PDFs estimated from the parents of ASD children (green traces). Bottom-right panel is elderly participants between 75 and 77 years old. **(B)** Estimated PDFs from neuropsychiatric and neurological disorders: top-left is ASD (3–25 years old). Top-right is SZ (22–57 years old, see Table S1 in Supplementary Material for age break down). Bottom-left is mild PD and bottom-right is severe PD. All PDFs are plotted against those of the deafferented subject (yellow trace is from the condition with visual feedback and black trace from the dark condition.) **(C)** Power law relation (see text for fit exponent value and goodness of fit) on the log–log Gamma parameter plane between the estimated shape and the estimated scale parameters of the continuous Gamma family of PDFs. Each dot corresponds to an individual participant. **(D)** Notice the mean values taken across participants in each group whereby the ASD group falls apart with the highest noise and the value of the shape parameter tending away from the Gaussian distribution case.

The cross-sectional data under examination revealed that participants with no clinical diagnosis had different statistical signatures across ages, suggesting that even during typical development these signatures of fluctuations in motor performance may shift. Table S6 in Supplementary Material reports the ranges of the estimated summary statistics (first, second, third, and fourth moments), for the normalized PV index corresponding to the control participants grouped by age. The results of the pairwise comparisons of the medians of each moment, using the nonparametric Wilcoxon rank sum test, are also reported in Table S6 in Supplementary Material. Here, the young children (CT1a) aged 3–4 years old showed the highest levels of noise-to-signal ratio, but this was not significantly different from those of children aged 5–10 years old (CT1b). The mean value of the normalized PV index did differ significantly between the two groups of children (*p* < 0.01), whereby the older children were significantly faster on average. Figure 3A (leftmost top panel) shows the estimated PDFs of the two groups superimposed and contrasts them to those of the young college participants (CT2 aged 18–25 years old) in the right panel. Figure 3A also shows the PDFs for the CT3 groups (aged 30–57) superimposed on those of the parents of children affected by ASD in this study (green traces). The latter group ranged in age from 32 to 44 years old, overlapping with those of members of the CT3 group, yet their estimated PDF’s fall closer to those of the elderly group (75–77 years old). Indeed, the statistical comparisons reported in Table S6 in Supplementary Material revealed no differences between the ASD parents and the elderly group across all moments, despite the large gap in these groups’ ages. Both the elderly groups and the ASD parents move at comparable speeds on average and have comparable levels in the accumulation of noise (Figure S3B in Supplementary Material). Yet ASD parents and the elderly groups do show differences in the time range of reaching the peaks (Figure S3C in Supplementary Material). Specifically, the probability plots of the time to reach the PV shows these differences. When examining the normalized PV index, the parents line up with the deafferented subject under visual guidance and with the elderly participants. There in Figure S3B in Supplementary Material, one can appreciate that the elderly participants move slower than the other controls. Yet, when we examine their timing to reach the peak, the elderly participants fall within the ranges of the younger controls. This suggests that under similar timing scale, it is the noise in the distance traveled by the hand up to PV that most likely account for their bradykinesia. This is in contrast to ASD parents who move slower than controls under a timing scale that rather aligns with that of the subject without proprioception (Figure S3C in Supplementary Material). The ASD parents are as slow as the elderly participants, but in their case, both the distance traveled to the PV and the time to cover that distance are problematic.

The ASD parents significantly differed from age-matched controls in CT3 in terms of average movement speed and noise levels (*p* < 10^−6^). The summary statistics map for all control participants, along with their median values, are shown in Figure S3A in Supplementary Material accompanying Table S6 in Supplementary Material. Likewise, the probability plots comparing all control groups are shown in Figures S3B,C in Supplementary Material. Notice the overlap of the parents’ signatures with those of the elderly controls (Figures 3C,D), as well as the separation between the deafferented subject and the CT3 control group who are of a similar age as this subject. CT2 is the ideal group in the sense that their distribution is normal (along the line of unity in the probability plot).

The lowest levels of noise-to-signal were registered in the young-to-mid age controls of the CT2 and CT3 groups. Figure 3C shows an emergent power relation between the estimated Gamma shape and scale parameters with model *f*(*x*) = *a* × *x^*b*^* common to all groups (fit with 95% confidence bounds), where *a* = 0.794 (0.747, 0.841)*; b* = −1.031 (−1.043, −1.019) and goodness of fit: sum squared error: 9.433e−07; adjusted *R*-squared: 0.9982; root mean squared error: 9.57e−05.

**Figure 4:**
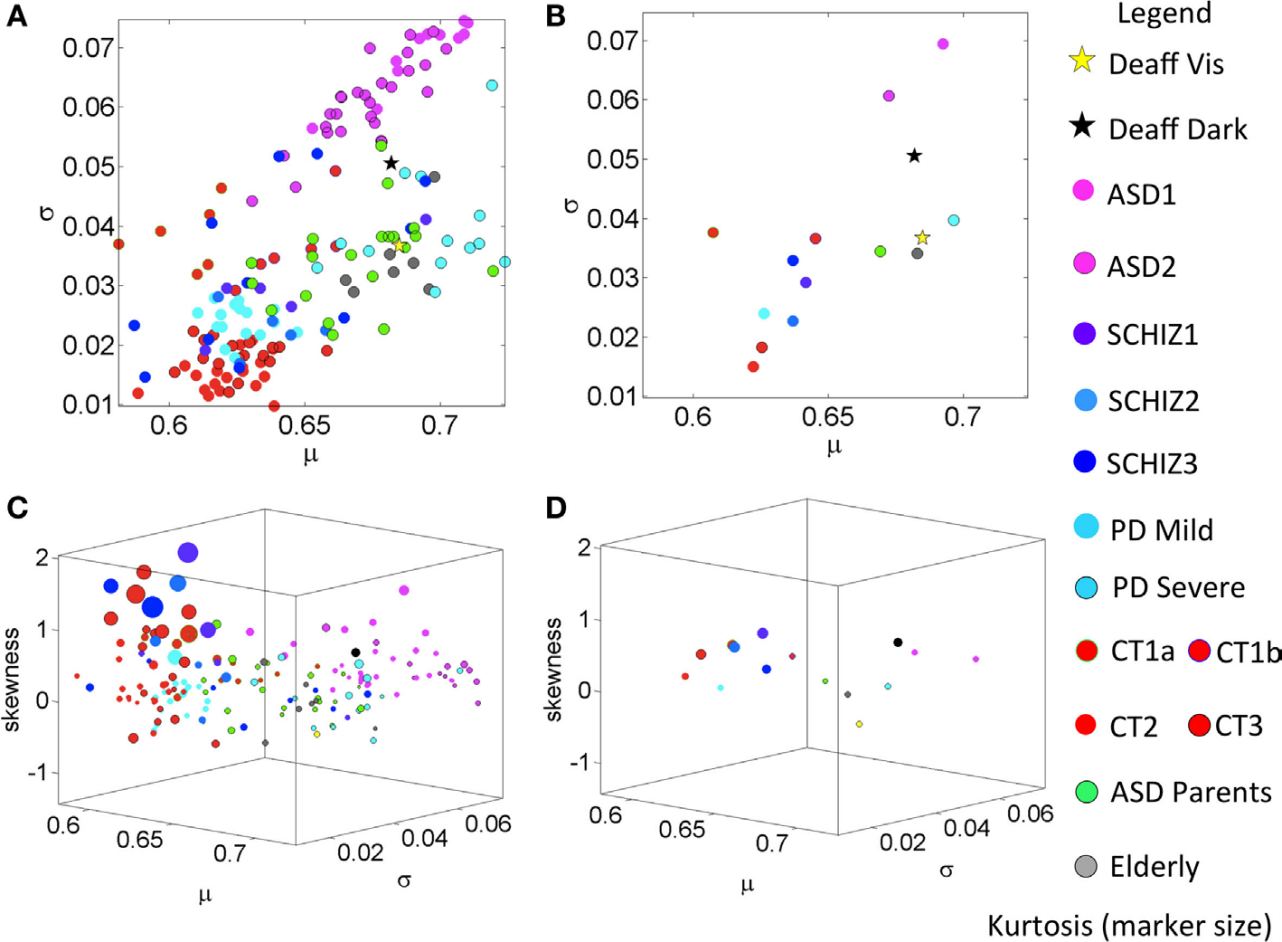
**Summary of empirically estimated statistical parameters across all conditions**. **(A)** Parameter plane spanned by the estimated mean and estimated variance across groups (see legend). **(B)** The mean values of each group. **(C)** Four-dimensional plot with skewness along the *Z*-axis and kurtosis represented by the size of the marker. The marker color defines the group type in the legend. **(D)** Mean values from each group.

All participants fall on this line with CT2–3 having the lowest noise-to-signal (scale) levels and the largest shape values – indicating distributions tending toward the Gaussian shape. The average parameter values per group are shown in Figure 3D. The middle-aged participants in CT3 showed the largest kurtosis values (see Figure 4C). Figure 4D summarizes the mean for each group, showing as well the shifts of these statistical signatures with normal development and aging. Along this map, the surprising finding was the overlapping of the signatures of the ASD parents with those of the elderly participants and away from those in age-matching CT3.

### Highest Speed-Dependent Noise in ASD Is Accompanied by Low Average Speed Values

Across all groups with neuropsychiatric/neurological disorders and the control groups, the ASD group generated the lowest values for the shape parameter and the highest values of the noise-to-signal ratio along the scale axis for the normalized PV index under examination.

**Figure 5:**
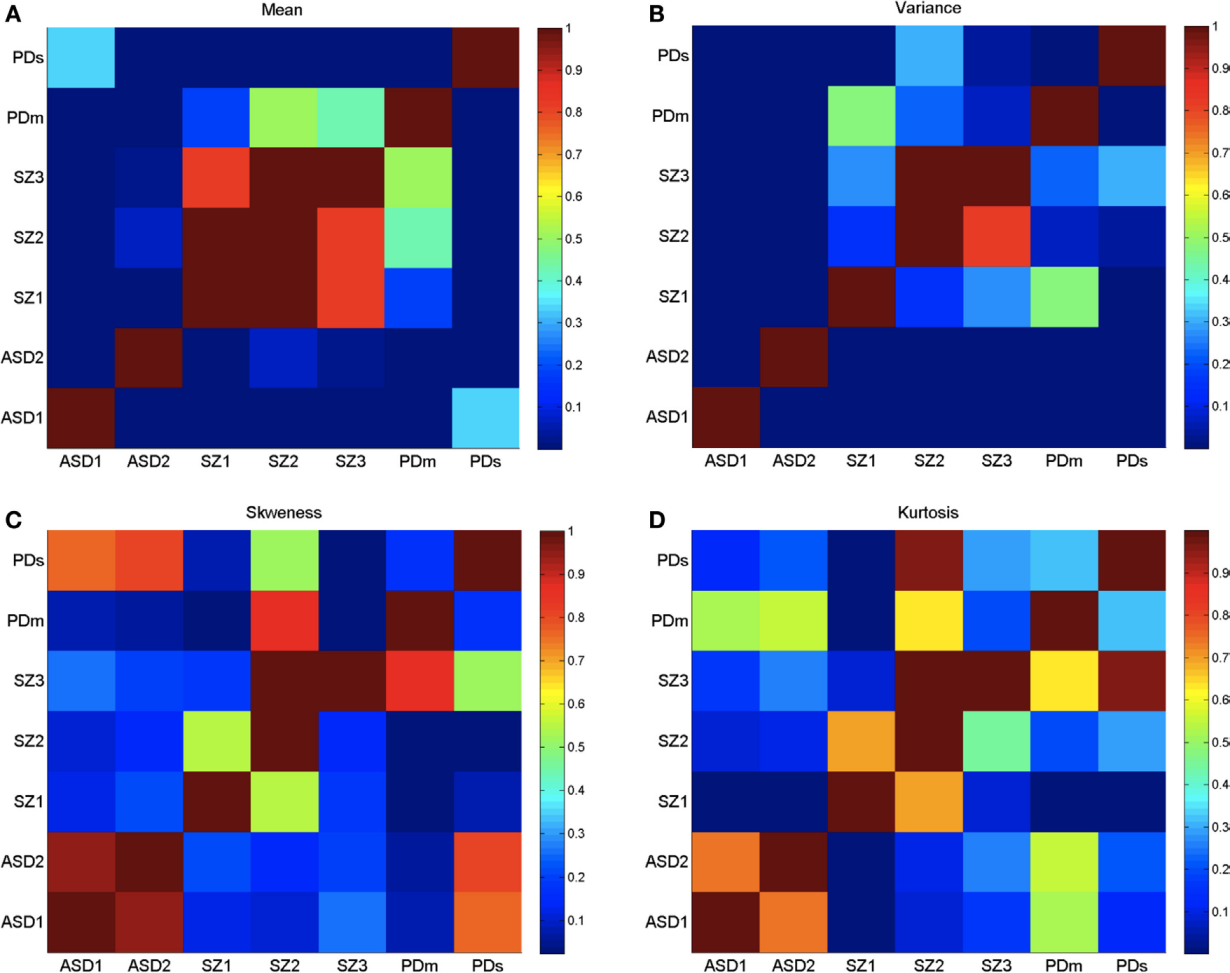
**Log of *p*-values from the non-parametric Wilcoxon ranksum test performed on the four moments of the empirically estimated PDF, taken pairwise across neurological conditions as divided by age (ASD and SZ) and severity (PD)**. **(A)** Empirically estimated mean. **(B)** Empirically estimated variance. **(C)** Empirically estimated skewness. **(D)** Empirically estimated kurtosis. Values of *p* > 0.05 are not statistically significantly different (ignore diagonal). Color bar are reflecting 10^−number^, the log of the *p*-values.

Figure 3B shows the empirically estimated PDFs with those of the deafferented participant superimposed (yellow and black traces, see legend). Figure 3C shows that all ASD subjects have higher noise, even more so than the deafferented subject, whether pointing in the dark or using visual feedback. The ASD highest variability level is shown in the two-dimensional plot of Figure 4A. The averaged summary statistics of these parameters in Figures 4B,D also demonstrate this. These participants also move at the slowest rate regardless of age. Further information about this group can be seen in the four-dimensional plot, in which the skewness and kurtosis of their empirically estimated distributions are also shown. There the performance of the deafferented participant, while pointing in the dark, falls within the distribution ranges of the ASD participants, specifically closer to those in the younger ASD subgroup. This can also be seen in Figure S4 in Supplementary Material accompanying Table S7 in Supplementary Material with a focus on the ASD cohort. For clarity, this figure isolates the ASD participants in relation to the controls of similar ages. Very little overlapping between controls and ASD can be detected in this parameter space. Table S7 in Supplementary Material provides the ranges and outcome of statistical comparisons, whereas Figure 5 provides a summary in matrix form of the *p*-values from pairwise comparisons.

Table S7 in Supplementary Material reveals significant statistical differences (*p* < 0.01) across all pairwise comparisons of noise levels between both ASD groups and controls subgroups with overlapping ages. This was also the case for the estimated mean with the exception of ASD1 and the elderly participants, and ASD2 and their parents.

Figure 3B also shows the largest dispersion for ASD across the cohort of patients and controls – comparable to the deafferented participant in the dark condition (black curve). However, the ASD group shows a mean value of the index that is shifted to the left, indicating faster movements on average than the deafferented participant (recall the denominator involving larger average speeds leads to a left shift of the index toward smaller values). In the ASD groups, as the average speed decreases (i.e., the normalized PV index increases), the variance increases.

### Parkinson’s Disease Patients Have Speed Statistics Comparable to Those of ASD Parents

A surprising result in the analyses of the typical controls was that ASD parents, who are young to middle aged, showed statistical features of the elderly group. Here, we also found unexpected similarities in the noise levels between the young ASD parents and the severe PD patients. The empirically estimated PDFs of the parents are seen in Figure 3A in the bottom-left panel, superimposed with the CT3 group of comparable age. Notice the disparity in dispersion between these groups; indeed the parents of children with ASD show results that are comparable to the elderly group of healthy adults. A further graphical view of this result is shown in Figures 3C,D, which illustrate the scatter across all participants and the groups’ averaged shape and scale parameter planes respectively. Figure 4A shows the scatter of the summary statistics for the ASD parents, overlapping mostly with the elderly and the severe PD participants. In Figure 4B, the average values taken across these three groups (elderly participants, ASD parents, and severe PD) are localized next to that of the deafferented participant pointing under visual feedback. Figures 4C,D show the 4D plots of the scatter and averaged values, with an additional plane, lifting the points according to the empirically estimated distribution of skewness and kurtosis (the size of the marker). This graphical illustration indicates that the signatures of the normalized velocity index of the ASD parents fall within the ranges of the elderly and severe PD patients, but farther away from the deafferented participant. In all plots, the ASD parents are not overlapping with the CT3 group who are within their age range. Figure S5C in Supplementary Material also shows a difference between the timing of the reaches of the ASD parents and the age-matched CT3. The probability plots of the time to reach the PV are shown in this figure for the ASD parents. They align with those of the mild PD patients and the deafferented subject under visual guidance. In contrast, the severe PD patients align their timing with that of the deafferented subject pointing in the dark.

### Summary Statistics Unambiguously Separate PD Subjects with Different Clinical Severity Levels

Table S3 in Supplementary Material reports the demographic and clinical information concerning the PD patients. Table S8 in Supplementary Material reports the ranges for the estimated statistical summary for PD patients of severe and mild-to-moderate stages according to clinical scores in Table S3 in Supplementary Material. These subgroups differ significantly in noise levels, estimated mean, and estimated variance of the normalized PV index (all rank sum tests on the medians *p* < 0.001) according to the empirically estimated distributions. The estimated PDF’s for the severe and mild PD groups are shown in Figure 3B lower panels (see legend). Note the broad dispersion of the severe group in contrast to the sharper PDFs of the mild-to-moderate group. These differences in mean and variance are also visible in Figure 3D summarizing the mean values of the estimated Gamma shape and scale parameters.

The mild PD patients are closer in speed to the middle-aged CT3 group (nonsignificant differences in mean value) and to the younger CT2 group (nonsignificant differences in mean value and skewness level). Thus these patients are not yet bradykinetic, but their noise levels are significantly higher than younger controls in CT2 and CT3 groups. The mild PD significantly differ (*p* < 0.001) from the ASD parents (Table S8 in Supplementary Material) but surprisingly, the severe PD cohort has comparable statistics in noise, variance, and skewness close to those of the much younger ASD parents and to the elderly controls between 75 and 77 years old. Note here again that the ASD parents are closer in age to the middle-aged CT3 group, much younger than these PD and elderly participants. Further differences between groups can be observed in the 4D plots of Figures 4C,D. Here, one can see that the mild-to-moderate PD group falls closer to controls than to the severe PD. The latter falls closer instead to the deafferented participant when he moves aided by visual guidance.

The estimated PDFs for the severe PD in Figure 3B (bottom right) show the overlap with the PDFs of the deafferented participant without vision. Likewise, Figure S5 in Supplementary Material accompanying Table S8 in Supplementary Material shows the summary statistics for these two groups of PD patients in relation to age-matched controls and to the ASD parents. Notice as well the probability plots whereby the probability distributions of the controls tend to normal (close to the line of unity), while those of the patients deviate from the line of unity.

### Statistical Ranges of SZ Patients Show Heterogeneity Relative to Other Neurological Disorders

Patients with SZ did not significantly differ in stochastic signatures across ages. This is shown in matrix form in Figure 5 for all moments. Furthermore, the younger SZ patients (22–30 years old) are closer to middle-aged CT3 adults than to young CT2 adults, despite overlapping ages with the latter group. Table S9 in Supplementary Material reports the ranges of all estimated signatures along with the statistical comparisons. Note that the estimated means of CT2 and CT3 do not significantly differ from SZ1, but the older SZ2 and SZ3 have noise levels comparable to those of the ASD parents and the elderly controls. Figure S6 in Supplementary Material, corresponding to Table S9 in Supplementary Material, shows the scatters of CT2, CT3, SZ1–3, and those of the ASD parents and the elderly group, all in relation to the deafferented participant pointing in the dark and pointing with visual guidance. Despite the overlap between some SZ and ASD parents, as well as the overlap of some SZ with the elderly participants, the average SZ groups stand on their own with the highest kurtosis in the estimated distributions. Figure S6 in Supplementary Material also shows the probability plots of SZ as a group (including all ages) in relation to CT3. Notice the deviation from the line of unity indicating departure from the normal distribution. Further, using the chi-square goodness of fit test for each individual age subgroup yielded significant deviations from normality (*p* < 0.05, *p* < 0.01, and *p* < 0.01 for young SZ1, middle-aged SZ2, and older SZ3 patients, respectively). The separation between SZ statistics and those of the deafferented participant in both pointing conditions is also evidence in this plot. The difference between the subjects with SZ and the ASD parents is also shown (parents divided by sex – red females and blue males). In summary, the statistics of SZ patients are atypical, highly heterogeneous, and different from those of other disorders.

### Temporal Differentiation between Forward and Retraction Movement Segments Differ across Neuropsychiatric/Neurological Disorders and Typical Cross-Sections of the Population

Examination of the normalized PV index, as a function of the proportion of time to reach the PV for each group within the study, revealed differences between the forward and the backward segments of the pointing loop. These differences for the control groups CT1, CT2, CT3, elderly participants, and ASD parents can be appreciated in Figures S1A in Supplementary Material (forward) and Figure S1B in Supplementary Material (retraction with the shift from the forward case). As the parents of individuals with ASD demonstrate the largest shift, we divided the group by sex into mothers and fathers. The legend provides information on the group type.

In this graph, along the *x*-axis, we plot the proportion of time to reach PV. Along the *y*-axis, we plot the normalized PV index. Typical ranges along the horizontal axis are about 0.5, where the peak tends to occur (midway between the two local minima). Lower values indicate reaching the speed maximum earlier, whereas higher values indicate reaching it later.

The graph in Figure S1A in Supplementary Material shows a separation between the younger CT1–3 groups and the remaining subjects, whereby the latter are slower on average (with higher normalized PV index). Along the time axis, these younger participants fall near 0.5, while the elderly participants and the parents vary. The elderly participants reach the peak earlier than half-way to the pause, and in the parents the mothers are similar to the younger controls but the fathers reach PV later. Indeed, the fathers of this cohort showed the largest difference in the proportion of time to reach the PV between the forward and the backwards reaches. The retractions peaked much earlier and the value of the normalized PV index dropped. This indicates a much faster retraction (as with the deafferented subject) whereby the end effector is retracted under less control. Specifically, the fathers were the slowest of the cohort to reach the velocity peak during the forward reach phase, but the retraction phase was almost a jerky, nonsmooth motion, suggesting poor motor control of these spontaneous, uninstructed reaches. The signatures of the deafferented participant are plotted for the cases of pointing in the dark and pointing when aided by visual feedback. In the dark, the deafferented participant reaches the PV earlier and moves faster on average than when guided by vision. This is expected and consistent with his description of feedforward strategies to initiate the motion and rely on visual feedback to compensate for the lack of proprioception. Evidence for feedforward control in IW was also shown in a mirror drawing task (33).

In the parameter plane of Figure S1B in Supplementary Material, we also plot the shifts comparing the timing in forward and back movements of the participants. Here, the ASD parents have the largest shifts in the retraction movements, toward the 0.5 values of the proportion of time and toward faster average speed along the *y*-axis. They perform more similar to controls in the retraction than in the forward segment. IW also shows changes in these patterns, with shifts in the opposite direction for the two conditions that he performed. Notice that the youngest group of children and the elderly participants show no discernible shift between forward and backwards reaches in these plots. Figure S1 in Supplementary Material also shows the distributions of these two parameters per group along each axis.

Figures S1C,D in Supplementary Material show similar plots for the patient groups under study in relation to the deafferented subject. All patients have slower average speeds than controls but the mild PD group shifts toward typical levels in the retractions. The severe PD group is, as expected, the slowest and tends to reach the PV earlier than the mild PD group. The distributions (color coded as in the legend) also show the differences across groups.

Further analyses of the temporal features for each group were performed on the actual time to reach the PV. The results are shown for each group in Figure S2 in Supplementary Material. Here, the axes are not adjusted, so as to allow for greater appreciation of the shapes and dispersions of the histograms within each group and in the forward and back condition. Note that the differences in scale along the *x-axis* of the time to reach the peak would prevent clarity if all graphs were set to the same scale. The Gamma plane summarizes the estimated Gamma parameters for each group and set of conditions under analysis (forward and retraction). The legend shows the color-coded data corresponding to the frequency histogram (each data set comprises 1000 data points randomly selected from the estimated Gamma parameters of the entire set). The ASD group is by far the most skewed, consistent with previous analyses of their temporal kinematics while performing other tasks (8, 32). The probability plot in Figure S2B in Supplementary Material (right panel) accompanying Table S7 in Supplementary Material captures the departure from normality of this temporal parameter in the ASD cohort. Note that in the Gamma parameter plane of Figure S2 in Supplementary Material, the temporal stochastic signatures of the ASD group (located at [2, 0.2]) in the shape, scale parameter plane, separate from the controls their age (located at [7, 0.1]), but close to the signatures from the elderly participants (located at [2, 0.1]) and the SZ patients (overlapping with the elderly participants).

As with the normalized PV index, this graph also shows that control groups CT2 and CT3 have the lowest noise-to-signal ratio and the largest shape values tending toward the Gaussian ranges of the Gamma plane. In stark contrast the temporal signatures of the subjects with severe PD and the deafferented participant are located far from the ideal controls.

## Discussion and Suggested Further Steps

This work characterized the statistical ranges of velocity-dependent fluctuations in pointing performance across a large heterogeneous cohort of human participants. Besides characterizing the signatures of cross-sections of the typical population across the life span, such signatures were also empirically estimated for individuals with neuropsychiatric and neurological disorders. These included neurodevelopmental (ASD) and neurodegenerative (PD) disorders. We also studied the performance patterns of patients with SZ, a syndrome with onset of its characteristic set of features (i.e., psychotic symptoms) in early adulthood (in contrast to ASD and PD), and for which very little motor control research exists. Finally, we included a deafferented participant who lacks proprioception due to damage in afferent fibers conducting touch, pressure, and movement information from the periphery to the brain.

The main purpose of this work was to estimate statistical ranges across the population so as to initiate a path of change in statistical analyses from the current “one-size-fits-all” model, to a more personalized approach in line with current NIMH initiatives. Here, we have shown that the typical patterns differ across different groups within the population, with ideal Gaussian-like shape and the lowest noise-to-signal ratio in the control subjects from college to middle age. The younger children and the elderly participants at the extreme of the bell curve of the human life span have different distribution shapes and dispersion that give rise to different summary statistics. In contrast to the ideal statistics from the young typical controls, the ASD, PD, SZ, and the elderly groups had marked differences along at least one of the moments’ axes that we empirically estimated here. Such differences were detected (Tables S6–S9 in Supplementary Material) above chance across the clinical groups.

In addition to the grouped data analyses, we also showed that the summary statistics spaces localize each individual with respect to other individuals. Without color coding the scatter by disorder or age, we can see a gradient of differences with clear separation between ASD and controls. Likewise, we can see a separation between mild and severe PD that is quite unambiguous. However, the SZ patients were more heterogeneous than the other groups with brain disorders, as can be seen in Figures 4A,C, where overlap with other groups is evident. They are mixed with the elderly participants, the middle-aged CT3, the ASD parents, the mild-to-moderate PD, and the severe PD groups. Two SZ patients fell at the tail of the ASD cluster. Of note, we know that other kinematic parameters unambiguously separate SZ from matched controls (Nguyen et al., under review^1^), which suggests that including more dimensions in the data representation across disorders would be more illuminating than projecting all information on one plane, or restricting our analyses to one set of parameters. Likewise, there is an imperative need to report psychotropic medication intake to researchers who study motor control in order to assess motor variability as a function of dosage, time under medication, and medication combinations, among other factors. It is broadly reported that psychotropic medications are known to have variable side effects on movement patterns. In the present cohort whether or not patients were on medication, motor noise signatures were different from those of controls. Yet the present personalized methods allow more detailed analyses based on medication status. This is an important additional dimension that needs to be explored at the individual level in future analyses.

The velocity-dependent parameters used here could unambiguously detect differences in levels of severity in PD and unique levels of noise in ASD. Regardless of age, sex, or medication status, these patterns were distinguishable in this cohort when labeling the locations by clinical condition. Likewise, taken as a group, the SZ had radically different distributions from the groups with overlapping ages, CT2, and CT3. At the individual level though, the data from the SZ patients also emphasize the need for a personalized approach to this devastating yet heterogeneous disorder. In this sense, the case of PD is relevant as it shows that the clinical diagnosis already does a good job at characterizing the emergence of relatively homogeneous subgroups as the disease progresses. The utility of these analytical tools in ASD has yet to be clinically confirmed as at present the diagnosis does not include motor symptoms at all. Likewise in ASD and SZ, it will be important to ascertain the effects of medication intake on motor patterns, a task that is now possible with this statistical platform.

A surprising result emerged from the data on ASD parents. Specifically, the signatures of ASD parents did not match those of typical controls their age. Instead, their signatures matched those of the elderly and the PD groups. There are many possible reasons and combinations of reasons for this result that we shall investigate in future work. One reason could be due to overall parental stress levels. Other reasons could include symptom-based medication intake (e.g., antidepressants, stimulants, etc.) and/or genetic predisposition. Given their much younger ages than the elderly and severe PD groups, it may be useful to study the rate of change of these patterns in ASD parents. In particular, tracking the evolution of the motor signatures with age may illustrate whether the signatures of their motor patterns are in an atypical accelerated state of change.

These data sets provide insights into the general motor statistics of these populations, and underscore the need for a personalized medicine approach to psychiatric and neurological disorders. In particular, the case of PD, a disorder that is diagnosed and tracked based on visible changes in motor patterns, beautifully illustrates the potential utility of motor noise-based biomarkers to characterize each person relative to the rest of the population. The two cohorts of PD, mild-to-moderate and severe, could serve as anchors to reference other more heterogeneous disorders such as SZ. Some of the SZ patients had patterns comparable to those of severe PD while others fell closer to those of the mild-to-moderate PD group, and yet others were overlapping with CT3 participants. Statistical distance metrics based on this type of sensory–motor noise may help us discern alterations in motor feedback as a function of anxiety, dopamine receptor-blocking medications, and other factors in these populations.

One of the most striking features in the data from subjects with SZ, besides its heterogeneity in the motor domain, is the lack of similarity with the deafferented participant. Unlike the ASD and severe PD groups, who were close to the deafferented participant pointing in the dark, the SZ patients fell far from IW in the statistics parameter space. Subject IW lacks feedback from the fibers that transmit touch, pressure, and movement information but has temperature and pain channels spared. The result is noteworthy given the reports in the SZ literature of problems with thermoregulation (34–36) and higher thresholds for pain perception across patients relative to controls (37–40). It will be interesting to investigate velocity-dependent motion parameters as a function of those autonomic signals in SZ. The present results demonstrate that proper statistical analytics applied to continuous recordings are required to provide more meaningful answers to basic research questions and establish the nature of the relationships between these afferent inputs and specific sensory–motor deficits in anticipatory behavior and volitional control.

In the context of internal models for action (IMA), it is possible that across these neurological disorders there are differentiable and selective disruptions in various components of forward planning linked to different levels motor noise. Among brain areas that are thought to be important for forward computations within the framework of IMA are the cerebellum (41, 42) and the posterior parietal cortex (PPC) (43). The cerebellum is known to be a problematic brain structure in all of these disorders (44–51). Likewise, connectivity issues between parietal and motor cortices have been reported in all these patient types. In SZ, this has been the case (52, 53). In ASD, connectivity problems are also reported (49), and in PD, striatofrontal regions seem to be affected (54) possibly impacting parietofrontal loops involved in forward planning and decisions. Notwithstanding issues with imaging studies (55), in the light of problems with the velocity-dependent signals that we have quantified here at the motor output level, it is possible that communication between these key nodes of the brain and the periphery may be corrupted by excess motor noise partly impeding the continuous afferent and reafferent flows from the periphery and possibly disabling predictive coding.

Velocity-dependent peripheral input signals from self-produced, goal-directed motions are an important source of guidance to the brain. They help compensate for synaptic transductions and transmission delays. In the context of visually guided reaching, areas in the PPC are known to be important for the planning and execution of such actions. Regions in the PPC receive eye position and velocity afferent inputs via ascending prepositothalamocortical pathways (56). Proprioceptive inputs required for proper visuomotor geometric transformations for reaches (57) have also been found to converge to the PPC (58) from the dorsal column nuclei and the postcentral somatosensory cortex. Given the putative roles of the PPC in forward prediction (43, 59, 60), trajectory formation (61–63), and geometric visuomotor transformations (64, 65) along with its cerebellar inputs to the lateral and medial intraparietal (LIP and MIP) cortical areas (66), we suggest that the motor–PPC–cerebellar networks may be selectively disrupted across these disorders, and that part of this disruption is due to poor continuous updating involving afferent sensory guidance from more than one sensory–motor channel.

Afferent sensory channels convey reafferent kinesthetic signals from mechanoreceptors involving touch, pressure, and ongoing self-produced movements. They also convey pain signals from nociceptors and temperature-related signals from thermoreceptors (67). The present work identifies interference with kinesthetic reafference from ongoing movements, but it will be important to examine afferent deficits concerning thermoregulation and pain perception, as these contribute to corporeal self-awareness. Corporeal self-awareness is critical in forward computations and geometric transformations bound to be disrupted in the face of excess motor noise found here in all disorders.

The new analyses reveal striking statistical differences between mild-to-moderate and severe stages of PD. In particular, these two cohorts have selectively overlapping features with the deafferented subject IW. In the case of the mild PD, the signatures overlap with those of IW under conditions of visual guidance. Interestingly, an overreliance on visual feedback has been reported in PD (7, 68–70), along with a new view that proprioceptive coordination may become impeded as the disease progresses (71, 72). In a previous study, an egocentric frame of reference for visual guidance (anchored at the moving finger), but not an allocentric frame of reference (anchored at the external target), helped mild PD patients improve many aspects of their pointing trajectories (7). Vision alone is not useful to the patients with mild PD, but vision aligned with self-generated motion shifts their movement statistics to typical ranges (7). Given the statistical similarity of the mild PD and IW with vision, it is possible that in mild PD patients, the signatures of reafferent minute motor fluctuations that we found to be corrupted by noise and randomness may improve when guided by vision. In the case of severe PD, their signatures rather overlapped with IW as he pointed in the dark. This is also interesting as timing in their bradykinetic motions was comparable to those of the ASD parents. This was a rather surprising finding given the age disparity and the lack of any kind of neurological diagnosis. This result suggests further study of familial ASD.

Traditional studies of motor control assume normality in the distributions of kinematic parameters. This work shows that there is a range of skewed distributions, from the memoryless exponential to the symmetric Gaussian in the velocity-dependent code of pointing behaviors. This result underscores the importance of providing an empirical characterization of the statistical properties underlying human movements. By assuming normality and smoothing out as noise the motor output fluctuations, we miss important information in the data from both typical and pathological conditions. This work also highlights the significance of individualized statistical assessments that may enable the discovery of self-emerging patterns inherent in the data. Analyzed as an ensemble using clinical labels, the data are very revealing when empirical statistical estimation is used, rather than theoretical assumptions and homogeneous treatment of the data. The clinical literature of motor control makes a number of assumptions that may blur the true features of the kinematics data from neuropsychiatric and neurological disorders. This work emphasizes the importance of reconsidering those traditional practices and researchers teaming up with clinicians to better inform data-driven approaches.

### Implications of the Characterization of Motor Noise for Genetics Research

We have demonstrated here the importance of providing empirical estimation of the statistical features underlying motor behaviors. Across different disorders of the nervous systems, we were able to characterize the ranges of statistical parameters that are traditionally treated as homogeneous under the assumption of normality in the movement data. The noise that is traditionally smoothed out through data averaging and the retracting movement segments that are often discarded as nuisances in the data revealed fundamental differences across neurological disorders that may be of use to genetics research. Specifically, classification of different types of sensory–motor noise may be possible and may aid in linking specific genetic factors that give rise to selectively different levels of synaptic noise with differentiable levels of sensory–motor noise. The specificity of these biometrics has yet to be tested, and better instrumentation discerning motor from sensory noise in electromyography combined with high dimensional kinematic signals will be required. We need to unveil the origins of synaptic noise in the first place before understanding different gradients of sensory–motor noise. Yet, the same personalized statistical platform presented here can be used to examine time series of other related signals. In disorders of known etiology, it should be possible to investigate these questions so as to build similar statistical maps to those presented here, whereby genetic factors and their resulting synaptic noise would be another data dimension. Such questions can be addressed using the present statistical platform.

As presented here with the deafferented subject in different contexts, we could assess the patterns of sensory–motor noise from individuals that go on to receive a diagnosis of ASD, SZ, or PD but for whom a genetic history is available. One such a group is the Fragile X-related disorders, where premutation carriers may receive an ASD diagnosis at an early age or a PD misdiagnosis at a later stage in life, or a diagnosis of mood and other psychiatric disorders in the case of female premutation carriers (73). We suggest a new research program linking these disorders and deafferentation whereby the same statistical platform that we term “precision phenotyping” in this work could be used to better characterize this family of disorders in the human population at large. In this sense, the present results may be an important step toward developing a new analytical platform for Precision Psychiatry.

## Author Contributions

Given the diverse population and clinical expertise, the following breakdown of contributions is in order: ET conceived study, analyzed all data, and wrote paper. Autism: RI, ET, and CW collected data; RI, CW, JN, and JJ designed study/analyses; and JN performed clinical assessment. Parkinson: RI and ET collected data; JS performed clinical assessment. Schizophrenia: JN and TP collected data and designed study/analyses; SS performed clinical assessment. Deafferented subject: JC collected data and performed clinical assessment. All authors participated in the editing and approval of the final version of the manuscript.

## Conflict of Interest Statement

The authors declare that the research was conducted in the absence of any commercial or financial relationships that could be construed as a potential conflict of interest.

## Footnotes

^1^Nguyen J, Majmudar U, Papathomas TV, Silverstein SM, Torres EB. Schizophrenia: the micro-movements perspective. (under revision, Neuropsychologia, 2015).
