# Supplementary material for: Toward Precision Psychiatry: Statistical Platform for the Personalized Characterization of Natural Behaviors
Source: Front Neurol. 2016 Feb 2;7:8. doi: 10.3389/fneur.2016.00008 (PMC4735831; doi:10.3389/fneur.2016.00008)
Supplement: Supplementary file 1 [file Data_Sheet_1.DOCX]

Supplementary Material

**Towards Precision Psychiatry: Statistical Platform for the Personalized Characterization of Natural Behaviors**

Elizabeth B Torres^*^, Robert W. Isenhower, Jillian Nguyen, Caroline Whyatt, John Nurnberger, Jorge V. José, Steven Silverstein, Thomas V. Papathomas, Jacob Sage, Jonathan Cole

*** Correspondence:** Elizabeth Torres, Psychology Department, Rutgers University, 152 Frelinghuysen Road, Piscataway, NJ 08854, USA.

[ebtorres@rci.rutgers.edu](mailto:ebtorres@rci.rutgers.edu).

# Supplementary Figures and Tables

The following figures supplement the results reported in the main text and the tables reporting the outcome of statistical tests. Tables 1-4 provide demographic information. Tables 5-8 provide statistical results.

## Supplementary Figures


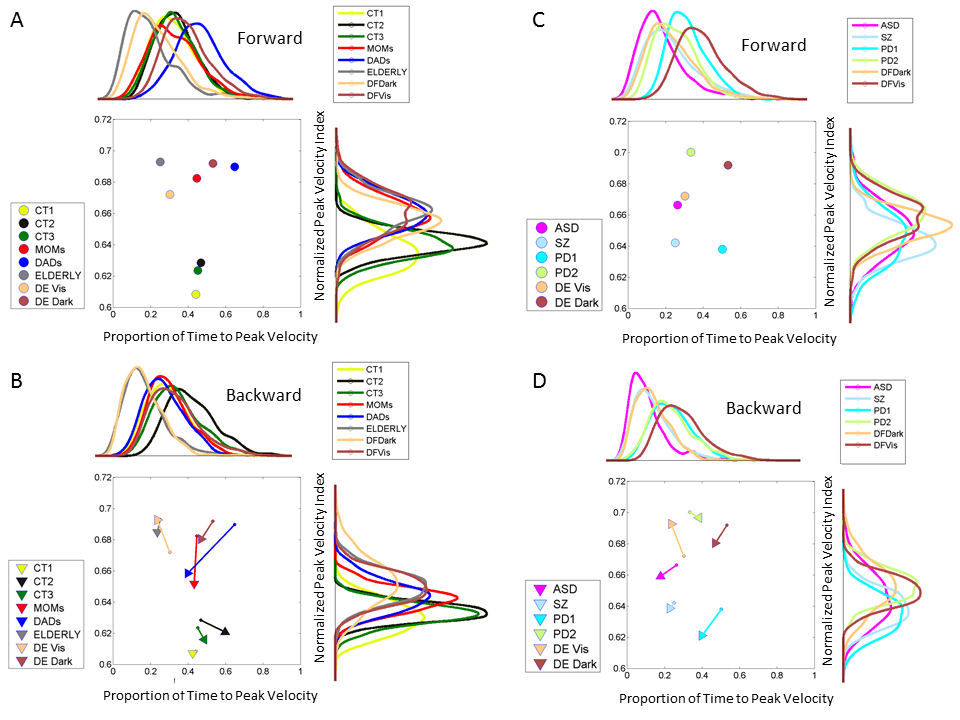


## Supplementary Figure 1: Scatter plots across controls and patients of the medians per group of the normalized peak velocity index (vertical axis) as a function of the proportion of time to reach the peak velocity (horizontal axis). Accompanying histograms built across 500 measurements randomly selected per group according to the empirical Gamma signatures of the ensemble data. (A) Signatures of forward reaches for controls of various age groups. (B) Signatures of backwards reaches indicating the change from the locations in (A). Notice that the children CT1 (3-10 years old), the college students CT2 (18-25 years old) and the middle age healthy controls CT3 (30-57 years old) group together on the parameter plane along the values denoting faster speed on average. The parents of children affected by ASD (32-44 years old) and the elderly (75-77 years old) group together along the locations denoting slower speeds. Plotted as well are the values of the deafferented subject (42 years old) who lost his proprioception from the neck down at 19 years of age. His motions were registered while using visual feedback (his default mode of movement) and also in the dark (forced to rely on motor imagery and the memory of the visual target). (C-D) The patterns from the patients are also shown for the forward (C) and backward movements (D). As in (B), the latter shows the shifts of the parameters with respect to the forward case.


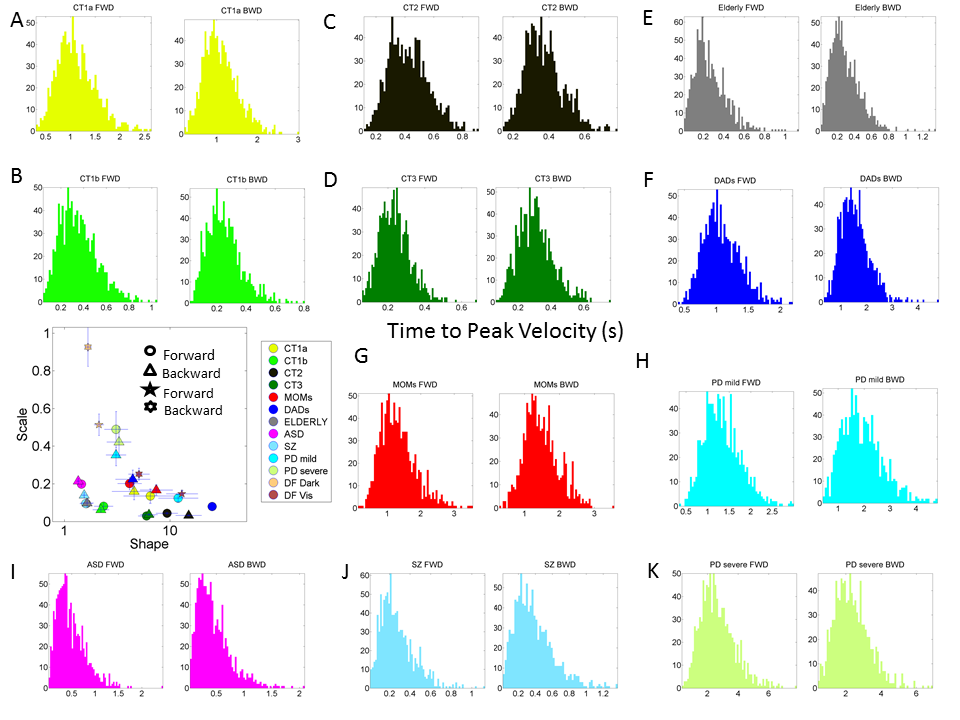


## Supplementary Figure 2: Histograms built from the time-period spanned from the movement onset to the time to reach the peak velocity for each group under study. (Corresponding stochastic signatures for the forward and backward reaches are also shown on the Gamma parameter plane). Each histogram comprises 1,000 measurements taken at random across each group according to the empirical Gamma values estimated from the ensemble data. Each panel has the forward and backward case plotted using the individual group’s range (for clarity). The Gamma plane summarizes the stochastic signatures empirically estimated from each forward and backward set. (A) Children between 3-4 years of age (CT1a). (B) Children between 5 and 10 years of age (CT1b). (C) Young controls 18-25 years old (CT2). (D) Controls between 30-57 years old (CT3). (E) Elderly group (75-77 years old). (F) Fathers of children affected by ASD (32-44 years old). (G) Mothers of children affected by ASD (32-39 years old). (H) Mild PD patients (58-79 years old). (I) ASD group (3-25 years old). (J) SZ group (22-57 years old). (K) Severe PD group (41-77 years old).

**
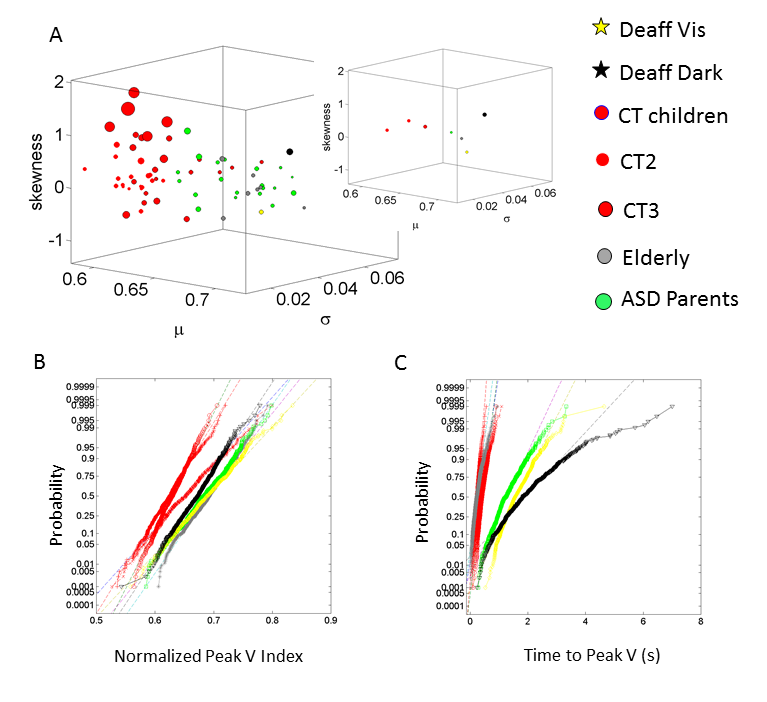
**

## Supplementary Figure 3 (supporting Table 6): (A) Summary statistics of the normalized peak velocity index compiled from all controls (see legend). Inset shows the mean values of each age group. Notice that the CT2 (19-25 years old) have the fastest speed on average, the lowest variance, and the closest to the symmetric distribution with kurtosis close to Gaussian ranges. They are the ‘ideal’ control group. Notice as well the evolution of these patterns over time whereby there is a shift towards higher variance and slower (bradykinetic) movements tending towards the signatures of the subject without proprioception (yellow marker) under overreliance on visual guidance for motor control. The ASD parents fall closer to the bradykinetic elderly with higher variability than age-matched CT3. (B) Probability plots highlighting the deviations of the distributions of the ASD parents, the elderly and the subject without proprioception from the age- and sex-matched controls. (C) The probability plots of the time to reach the peak velocity shows differences as well between the controls and the ASD parents. The parents line up with the deafferented subject under visual guidance and with the elderly. Here one can appreciate that the elderly participants move slower than the other controls, yet their timing are within the ranges of the younger controls, suggesting that under similar timing scale, it is the noise in the distance traveled by the hand up to peak velocity that most likely accounts for their bradykinesia. This is in contrast to ASD parents who move slower than controls under a timing scale that rather aligns with that of the subject without proprioception. The ASD parents are as slow as the elderly, but in their case, both the distance traveled to the peak velocity and the time to cover that distance are problematic.

**
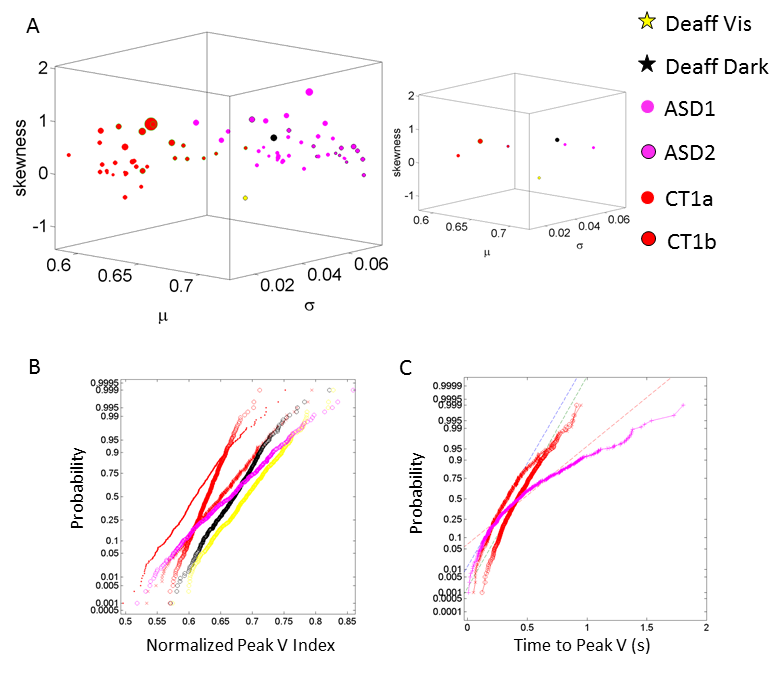
**

## Supplementary Figure 4 (supporting Table 7): (A) Summary statistics of the normalized peak velocity index for the ASD participants in relation to age- and sex-matched controls. Note that the ASD stand apart from age-matched controls that move faster on average and have lower variability. The inset shows the mean signatures of ASD children broken down into two main groups (3-12 years old and 13-25 years old). The latter falls closer to the signatures of the deafferented participant as he points in the dark. (B) Probability plots of the normalized peak velocity index from young CT1a,b and young college controls CT2, whose probability plot falls on the line of unity because the distribution approaches the normal distribution. (C) The probability plot of the time to the peak velocity from the ASD participants (pooled across all ages) in relation to CT1a and CT1b. Notice the deviation of this temporal parameter in ASD from the normal distribution (failing the normality test Chi-Square goodness of fit test P<1.3167x10^-7^, Chi-Square statistic 34.84.)


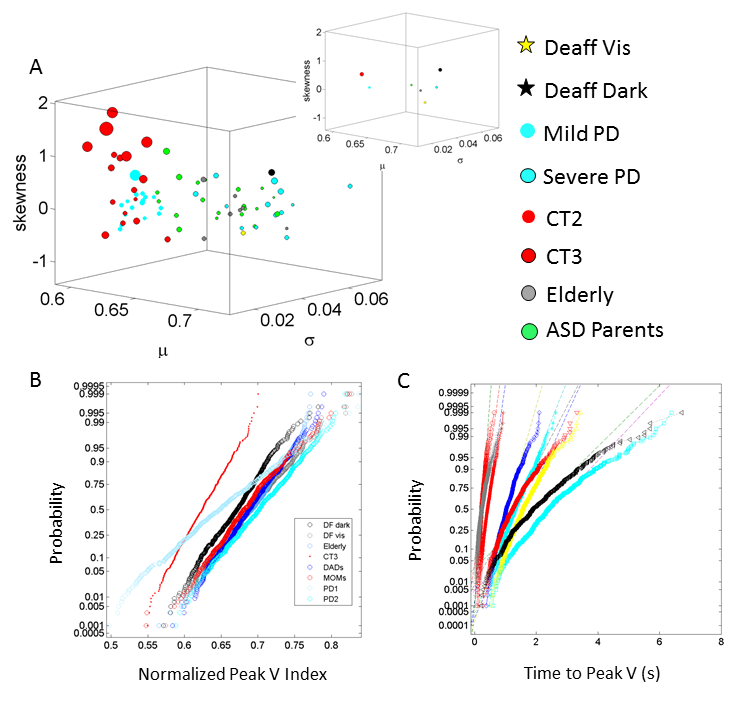


## Supplementary Figure 5 (supporting Table 8): (A) Summary statistics from participants with PD against age-matched CT3. Controls include as well ASD Parents 32-44 years old (green) and Elderly 75-77 years old (gray). Mild PD2 fall closer to CT3 but despite the age gap, the severe PD patients (labeled PD2) overlap with most of the ASD Parents. PD2 also overlap with the Elderly of comparable age and with the deafferented participant. (B) Probability plots of the normalized peak velocity index show the departure from normality in the data from the mild PD patients (labeled PD1), severe PD2, ASD parents (mothers in red, fathers in dark blue) and the deafferented participant in yellow (vision) and black (dark). Red dots are CT3 (middle age controls). The elderly’s probability plot (gray) also overlaps with those of the patients. (C) The probability plots of the time to reach the peak velocity reveal fundamental differences between controls (CT2, CT3, elderly) and the patients with PD. Severe PD patients align their timing with the deafferented subject as he points in the dark. Mild PD patients align their timing with the deafferented subject as he points guided by vision. Note that the ASD parents also align their timing statistics with the Mild PD patients and with the deafferented subject guided by vision.


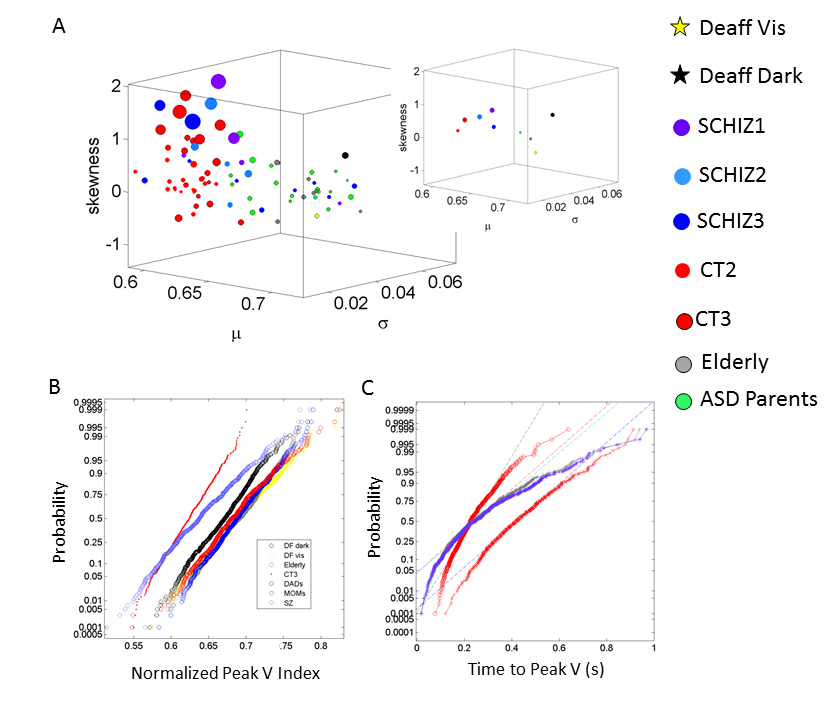


## Supplementary Figure 6 (supporting Table 9): Summary statistics of the normalized peak velocity index from participants with SZ compared to age-matched CT3. Controls include as well ASD Parents 32-44 years old (mothers in red and fathers in blue) and Elderly 75-77 years old (gray). The SZ cohort was further subdivided by age (SZ1, SZ, SZ3) from younger in their early twenties to older in their late fifties early sixties. The younger participants SZ1 (22-25 years old represented in light blue) overlap with ASD parents and some of the CT2, but the rest overlap with some age-matched CT3 and have on average much higher kurtosis (peaky distributions) than controls. Only a few overlap with the elderly and their signatures fall far from those of the participant without proprioception. (B) Probability plot sets the SZ group (pooled across all ages) apart from the controls (CT3) and also from the other controls (ASD parents and the elderly). They also separate from the participant without proprioception according to this index. (C) Probability plots of the time to reach the peak velocity separate the SZ patients (pooled across ages) from the CT2 group and also from the CT3 group. The SZ patients align the timing of their reaches with the elderly controls. The elderly move slower than CT2-3 groups but we saw in Supplementary Figure 5 that they do not align with the PD or ASD parents whose timing ranges are in a different time scale.

## Supplementary Tables

## Supplementary Table 1. Demographic characteristics of the 173 participants

| **Subject Class** | **Number**  **total (sub-groups)** | **Ages (years)** | **Sex (male/female)** |
| --- | --- | --- | --- |
| ASD (ASD1, ASD2) | 39 (19,20) | 4-12, 13-25 | 29/10 |
| PD (PD1, PD2) | 26 (9,17) | 41-77 | 19/7 |
| SZ (SZ1, SZ2, SZ3) | 23 (5,8,10) | 22-30,31-40, 41-57 | 18/5 |
| CT(CT1a,CT1b,CT2,CT3) | 58 (6, 12, 22, 18) | 3-4, 5-10, 18-25, 30-57 | 39/19 |
| Parents (of ASD child) | 21 | 32-44 | 12/9 |
| Elderly | 8 | 75-77 | 5/3 |
| Deafferented | 1 | 42 | 1 |

## Supplementary Table 2: Scores from clinical assessments of the participants with ASD

Stanford-Binet 5^th^ edition was used to assess intelligence of each participant with ASD (Roid, 2003). A score of 100 is the norm and a departure by 15 points indicates one standard deviation above or below typical intelligence. *NVIQ* is a measure of nonverbal IQ. *VIQ* is a measure of Verbal IQ. *FSIQ* is the sum of verbal and non-verbal intelligence scores converted to a standardized score. ADOS (Autism Diagnostic Observational Scale) (Lord et al., 2000;Gotham et al., 2009) is a standard assessment tool used by clinicians as a basis for the ASD diagnosis. Module 1 of the ADOS was used for the young, non-verbal students. Module 3 was used for the adolescent students with conversation ability. *Stereo* is a measure of stereotyped behaviors where a higher score indicates more stereotyped behaviors; however without cutoff for a ASD diagnosis. *Com* is the total Communication score, where 4 is the cutoff for Autism and 2 the cutoff for Autism Spectrum. *Soc* is the total Reciprocal Social Interaction Score, where 4 is the cutoff for Autism, and 2 the cutoff for Autism Spectrum. *Com + Soc* is the combined Communication and Social Interaction score, with a score of 12 being the Autism cutoff, and 7 the Autism spectrum cutoff. Because of their age and extremely limited verbal ability, 2 children could not be given the ADOS. Therefore the GARS 2 (Gilliam Autism Rating Scale – Second edition) (Gilliam, 2006) was used to assess these individuals. *Stereo SS* is the standardized score of stereotyped behaviors. *Com SS* is the standardized score of Communication. *Social SS* is the standardized score of Social Interaction. The Autism Index is the sum of standard scores, converted to normed index score.

|  |  |  | **Stanford-Binet** | | | **ADOS Scores** | | | | **GARS Scores** | | | |
| --- | --- | --- | --- | --- | --- | --- | --- | --- | --- | --- | --- | --- | --- |
| **Code** | **Gender** | **Age (yrs)** | **NVIQ** | **VIQ** | **FSIQ** | **Stereo** | **Com** | **Soc** | **Com + Soc** | **Stereo SS** | **Com SS** | **Soc SS** | **Autism Index** |
| **01** | M | 4.3 | 42 | 43 | 40 | 4 | 8 | 13 | 21 | N/A | N/A | N/A | N/A |
| **02** | F | 5.9 | 44 | 51 | 45 | 2 | 4 | 13 | 17 | N/A | N/A | N/A | N/A |
| **03** | M | 6.0 | N/A | N/A | 100 | N/A | N/A | N/A | N/A | N/A | N/A | N/A | N/A |
| **04** | M | 6.3 | N/A | N/A | 50 | N/A | N/A | N/A | N/A | N/A | N/A | N/A | N/A |
| **05** | M | 7.6 | 50 | 46 | 45 | 3 | 6 | 11 | 17 | N/A | N/A | N/A | N/A |
| **06** | F | 7.8 | 42 | 43 | 40 | 4 | 7 | 12 | 19 | N/A | N/A | N/A | N/A |
| **07** | M | 7.8 | 42 | 43 | 40 | 2 | 6 | 14 | 20 | N/A | N/A | N/A | N/A |
| **08** | M | 9.0 | 42 | 44 | 40 | 1 | 5 | 10 | 15 | N/A | N/A | N/A | N/A |
| **09** | M | 9.9 | 42 | 43 | 40 | 4 | 5 | 8 | 13 | N/A | N/A | N/A | N/A |
| **10** | M | 10 | N/A | N/A | 107 | 3 | 3 | 9 | 12 | N/A | N/A | N/A | N/A |
| **11** | M | 10 | N/A | N/A | N/A | 4 | 5 | 6 | 11 | N/A | N/A | N/A | N/A |
| **12** | N | 10 | N/A | N/A | 107 | 3 | 4 | 9 | 13 | N/A | N/A | N/A | N/A |
| **13** | M | 10.3 | 42 | 43 | 40 | 3 | 4 | 10 | 14 | N/A | N/A | N/A | N/A |
| **14** | M | 11.5 | 100 | 82 | 90 | 7 | 5 | 6 | 11 | N/A | N/A | N/A | N/A |
| **15** | F | 11.5 | 50 | 43 | 44 | N/A | N/A | N/A | N/A | N/A | N/A | N/A | N/A |
| **16** | M | 11.7 | 42 | 43 | 40 | 5 | 8 | 10 | 18 | N/A | N/A | N/A | N/A |
| **17** | M | 11.7 | 43 | 43 | 40 | N/A | N/A | N/A | N/A | N/A | N/A | N/A | N/A |
| **18** | M | 12 | N/A | N/A | 67 | 4 | 5 | 13 | 18 | N/A | N/A | N/A | N/A |
| **19** | F | 12 | N/A | N/A | 60 | 4 | 8 | 10 | 18 | N/A | N/A | N/A | N/A |
| **20** | M | 12 | N/A | N/A | 95 | 2 | 5 | 8 | 13 | N/A | N/A | N/A | N/A |
| **21** | M | 12 | N/A | N/A | 95 | 1 | 5 | 7 | 12 | N/A | N/A | N/A | N/A |
| **22** | M | 13 | N/A | N/A | 89 | 2 | 3 | 7 | 10 | N/A | N/A | N/A | N/A |
| **23** | M | 13.8 | 42 | 43 | 40 | N/A | N/A | N/A | N/A | N/A | N/A | N/A | N/A |
| **24** | M | 14 | N/A | N/A | 74 | 3 | 9 | 10 | 19 | N/A | N/A | N/A | N/A |
| **25** | F | 14.3 | 50 | 43 | 44 | N/A | N/A | N/A | N/A | 8 | 11 | 9 | 124 |
| **26** | F | 15 | N/A | N/A | 52 | 2 | 6 | 11 | 17 | N/A | N/A | N/A | N/A |
| **27** | F | 15 | N/A | N/A | 77 | N/A | N/A | N/A | N/A | N/A | N/A | N/A | N/A |
| **28** | F | 15 | N/A | N/A | 71 | 6 | 5 | 7 | 12 | N/A | N/A | N/A | N/A |
| **29** | M | 15 | N/A | N/A | 56 | 3 | 4 | 10 | 14 | N/A | N/A | N/A | N/A |
| **30** | F | 15.8 | 42 | 43 | 40 | N/A | N/A | N/A | N/A | 13 | 10 | 11 | 109 |
| **31** | M | 16 | N/A | N/A | 100 | N/A | N/A | N/A | N/A | N/A | N/A | N/A | N/A |
| **32** | F | 16 | N/A | N/A | 81 | 2 | 7 | 9 | 16 | N/A | N/A | N/A | N/A |
| **33** | M | 18 | N/A | N/A | 101 | 2 | 4 | 6 | 10 | N/A | N/A | N/A | N/A |
| **34** | M | 18 | N/A | N/A | 96 | 4 | 4 | 8 | 12 | N/A | N/A | N/A | N/A |
| **35** | M | 18 | N/A | N/A | 76 | 1 | 5 | 7 | 12 | N/A | N/A | N/A | N/A |
| **36** | M | 18 | N/A | N/A | 76 | 1 | 5 | 7 | 12 | N/A | N/A | N/A | N/A |
| **37** | M | 19 | N/A | N/A | 101 | N/A | N/A | N/A | N/A | N/A | N/A | N/A | N/A |
| **38** | M | 20 | N/A | N/A | 70 | 1 | 5 | 6 | 11 | N/A | N/A | N/A | N/A |
| **39** | M | 25 | N/A | N/A | 99 | 6 | 3 | 7 | 10 | N/A | N/A | N/A | N/A |

## Supplementary Table 3: Information from PD participants

Demographic and clinical features of 26 PD patients tested in the “off” state using the UPDRS uniﬁed PD rating scale; motor subscale. Mild PD patients averaged 71.4 years old (ranging from 58 to 77 years old) whereas severe PD patients averaged 63.4 years old (ranging from 41 to 81 years old).

In some cases patients were studied on more than one day. UPDRS scores were then averaged. In some cases, the UPDRS examination was not done for all items. In this case, the score was normalized to a total possible score of 108.

[**Medications**](#_bookmark1) **(**Anti-parkinsonian and related medications): A, amantidine; Be, benztropine; Bu, buspar; C, clonazepam; E, vitamin E; L, levodopa preparation regular release; LS, levodopa preparation, sustained release; Lu, ludiomil; No, notriptyline; Pe, pergolide; Pra, pramipexole; Pro, propanolol; Ras, rasagiline; Rop, ropinirole; S, selegiline; Sy, synthroid; T, trihexyphenidyl; To, tolcapone; aUPDRS scores unavailable for this patient. 1Hoehn and Yahr stage; 2Maximum score 108; 3Refers to number of years since ﬁrst remembered Parkinsonian symptoms. DBS@ means Deep Brain Stimulation at age.

| **Subject** | **Sex** | **Age** | **Stage1** | **UPDRS2** | **Symptoms (years)3** | **Medicines** |
| --- | --- | --- | --- | --- | --- | --- |
| **1** | M | 73 | 2.0 | 24.7 | 25 | L,Per, Tri |
| **2** | M | 75 | 2.5 | n/a | 16 | E, LS, Pro |
| **3** | M | 74 | 2.5 | 47.8 | 10 | Be, L, S |
| **4** | F | 79 | 3.0 | 15.8 | 4 | A, L, Pra, S |
| **5** | M | 75 | 2.5 | 27.0 | 8 | Bu, C, Lu, S |
| **6** | M | 77 | 3 | 28.5 | 9 | none |
| **7** | M | 58 | 2.0 | 21.5 | 8 | L, LS, To, Pe |
| **8** | M | 72 | 3 | 43.5 | 5 | Pe, S |
| **9** | M | 58 | 2.5 | 30.1 | 4 | L, S |
| **10** | F | 64 | 3 | 28 | 4 | L |
| **11** | M | 41 | 2 | 15 | 2 | A; L |
| **12** | M | 57 | 3 | 29 | 4 | L; Pra |
| **13** | M | 49 | 3 | 42 | 6 | Ras; Rop; Tri |
| **14** | M | n/a | 3 | 31 | 3 | L |
| **15** | M | 80 | 4 | 42 | 9 | L |
| **16** | F | 67 | 3 | 31 | 5 | L; Ras |
| **17** | F | 77 | 4 | 35 | 6 | L; Pra |
| **18** | F | 55 | 3 | 27 | 6 | L; Ras; Rop |
| **19** | M | 77 | 3 | 31 | 7 | Don; L |
| **20** | M | 72 | 2 | 21 | 8 | L; Pra; Ras |
| **21** | M | 54 | 4 | 42 | 9 | L; Rop; Sel |
| **22** | M | 60 | 3 | 35 | 10 | A |
| **23** | F | 69 | 2 | 18 | 12 | L; Pra; Ras |
| **24(DBS@47)** | M | 52 | 3 | 27 | 14 | A; L; Pra |
| **25(DBS @48)** | M | 59 | 4 | 42 | 19 | L |
| **26(DBS @70)** | F | 81 | 4 | 38 | 20 | L; Pra |
| **Summary** | **7F/19M** | **71.2 ± 7.8** | **2.5 ± 0.4** | **29.8 ± 10.7** | **9.8 ± 6.7** |  |

## Supplementary Table 4: Demographic Information for SZ Patients

| **Subject** | **Sex** | **Age** | **Age at First Diagnosis^1^**  **Approx date** | **Patient Type** | **Medicines** |
| --- | --- | --- | --- | --- | --- |
| **1** | M | 22 | 20 (06/17/2013) | Partial | Paliperidone |
| **2** | M | 24 | 19 (06/30/2009) | Partial | Zyprexa; Klonopin; Lamictal |
| **3** | M | 26 | 25 (02/1/2014) | Partial | Fluoxetine; Latuda |
| **4** | M | 30 | 21 (05/28/1993) | Outpatient | None |
| **5** | M | 30 | 27 (01/16/1999) | Partial | Lithium; Clozaril; Amantadine; Klonopin |
| **6** | M | 32 | n/a | Partial | Gabapentin; Zyprexa |
| **7** | M | 32 | 14 (11/14/1997) | Partial | Clozaril; Risperdone; Depakote |
| **8** | M | 33 | 13 (06/24/1995) | Outpatient | Latuda; Klonopin |
| **9** | F | 35 | 30 (2010) | Outpatient | Latuda |
| **10** | M | 40 | n/a | Partial | Risperidone; Valproic Acid |
| **11** | M | 46 | 21 (01/01/1990) | Partial | Klonopin; Trazadone; Prolixin |
| **12** | M | 47 | 36 (2004) | Partial | Abilify |
| **13** | M | 47 | 28 (01/15/1996) | Partial | Depakote; Klonopin; Cogentin |
| **14** | M | 50 | 33 (10/20/1998) | Outpatient | Seroquel; Abilify |
| **15** | M | 52 | n/a | Partial | Haloperidol |
| **16** | M | 52 | 24 (11/21/1987) | Partial | Zyprexa |
| **17** | F | 53 | 21 (1983) | Outpatient | Zyprexa; Depakote; Paliperidone |
| **18** | F | 54 | n/a | Partial | Klonopin; Abilify; Cymbalta; Remeron |
| **19** | F | 55 | n/a | Outpatient | Risperdone, Cogentin; Seroquel |
| **20** | F | 55 | 25 (05/29/1985) | Outpatient | Latuda; Fluphenazine |
| **21** | M | 40 | n/a | Outpatient | none |
| **22** | F | 35 | 30 (2010) | Partial | Latuda |
| **23** | M | 30 | 14 (1/16/1999) | Partial | Lithium, Clozaril, Amantadine, Klonopin |
| **Summary** | **5F/15M** | **40.75 ± 11.47** | **23.80 ± 6.41** |  |  |

^1^ Self-reported date of first symptoms. Entry n/a means the patient cannot recall. Most patients recalled exact date while others only recall the year.

**Supplementary Table 5:** Frontal System Behavior Scale (FrSBE) self-rating form test scores for Executive Dysfunction

| FrSBE Score for Executive Dysfunction (Clinical Range: 65-130, Borderline: 60-64) | | |
| --- | --- | --- |
|  | Before Illness | Present Time |
| SZ Group (*n* = 18) | 72.39 +/- 20.89 | 64.11 +/-20.89 |
| SA Group (*n* = 5) | 54.25 +/- 22.70 | 59.5 +/- 17.14 |
| All Patients (*n* = 23) | 69.09 +/- 21.86 | 63.27 +/- 14.20 |

FrSBE scores: Patients were asked to rate each statement presented in the FrSBE form from a scale of 1-5 on how the given phrase, such as “I feel confused” applies to oneself, with 1=”Almost Never” and 5=”Almost Always”. Patients were asked to score themselves “Before Illness” and “At the Present Time”. A score of 65-130 is determined to be in the clinical range. Scores between 60-64 are considered borderline. (SA stands for schizoaffective).

## Supplementary Table 6: Summary statistics of the ranges of first, second, third and fourth estimated moments for the normalized peak velocity index for all the controls and their statistical comparisons using the non-parametric rank sum Wilcoxon test.

| **Controls per Age Group**  **Median and Ranges** | **Noise to Signal** | **First Moment Mean** | **Second Moment Variance** | **Third Moment Skewness** | **Fourth Moment Kurtosis** |
| --- | --- | --- | --- | --- | --- |
| **CT1a 3-4**  **Median**  **Min**  **Max** | 0.224  0.014  1.065 | 0.614  0.608  0.623 | 0.002  0.001  0.002 | 0.224  0.014  1.065 | 3.559  2.197  7.823 |
| **CT1b 5-10**  **Median**  **Min**  **Max** | 0.002  0.001  0.004 | 0.645  0.624  0.661 | 0.001 0.0009 0.002 | 0.427  0.271  0.920 | 2.925  2.828  4.273 |
| **CT2 18-25**  **Median**  **Min**  **Max** | 3.628 x10^-4^  1.481 x10^-4^  6.868 x10^-4^ | 0.6261  0.5885 0.6388 | 0.221 x10^-3^  0.094 x10^-3^ 0.433 x10^-3^ | 0.1162  -1.4287 0.8313 | 4.085  2.043 16.286 |
| **ASD Parents 24-42**  **Median**  **Min**  **Max** | 0.0019  0.0007 0.0042 | 0.676  0.630  0.719 | 0.001  0.0005 0.003 | 0.1629  -0.2275 1.0359 | 2.687  1.484  4.492 |
| **CT3 Age 40-57**  **Median**  **Min**  **Max** | 5.317 x10^-4^  2.367 x10^-4^  8.205 x10^-4^ | 0.6253  0.6020 0.6582 | 3.336 x10^-4^  1.472 x10^-4^  4.996 x10^-4^ | 0.682  -0.369 1.793 | 4.525  2.805  4.492 |
| **Elderly 75-77**  **Median**  **Min**  **Max** | 0.0015  0.0012 0.0033 | 0.6828  0.6652 0.6979 | 0.0010  0.0008 0.0023 | 0.2055  -0.4485 0.7103 | 2.8241  2.2789 3.7273 |
| **Controls Age Group**  **P value Rank Sum Test** | **Noise to Signal** | **First Moment Mean** | **Second Moment Variance** | **Third Moment Skewness** | **Fourth Moment Kurtosis** |
| **CT1a *vs*. CT1b** | 0.2403 | 0.0022 | 0.3095 | 0.3939 | 0.1797 |
| **CT2 *vs*. ASD Parents** | 1.0124 x10^-7^ | 8.1758 x10^-7^ | 1.012 x10^-7^ | 0.3761 | 1.254 x10^-4^ |
| **CT2 *vs*. CT3** | 0.002 | 0.7755 | 0.0026 | 0.0365 | 0.241 x10^-4^ |
| **CT2 *vs.* Elderly** | 1.360 x10^-4^ | 1.360 x10^-4^ | 1.360 x10^-4^ | 0.8623 | 0.004 x10^-4^ |
| **CT3 *vs*. ASD Parents** | 5.3499 x10^-7^ | 2.8957 x10^-6^ | 3.3124 x10^-7^ | 0.1314 | 1.4065 x10^-5^ |
| **CT3 *vs.* Elderly** | 1.788 x10^-4^ | 1.788 x10^-4^ | 1.788 x10^-4^ | 0.0987 | 0.0015 |
| **ASD Parents *vs.* Elderly** | 0.561 | 0.081 | 0.638 | 0.639 | 0.489 |

## Supplementary Table 7: Summary statistics of the ranges of first, second, third and fourth estimated moments for the normalized peak velocity index for the ASD participants across age groups and their statistical comparisons to controls using the non-parametric rank sum Wilcoxon test.

| **ASD per Age Group**  **Median and Ranges** | **Noise to Signal** | **First Moment Mean** | **Second Moment Variance** | **Third Moment Skewness** | **Fourth Moment Kurtosis** |
| --- | --- | --- | --- | --- | --- |
| **ASD1 4-12**  **Median**  **Min**  **Max** | 0.0074  0.0049 0.0081 | 0.6938  0.6527 0.7102 | 0.0052  0.0032 0.0056 | 0.2702  -0.1509 0.8553 | 2.7794  2.2961 3.8473 |
| **ASD2 13-25**  **Median**  **Min**  **Max** | 0.0051  0.0031 0.0059 | 0.6636  0.6306 0.6953 | 0.0034  0.0020 0.0040 | 0.2482  -0.0386 0.9353 | 2.8720  2.1971 3.9919 |
| **ASD per Age Group**  **P value Rank Sum Test** | **Noise to Signal** | **First Moment Mean** | **Second Moment Variance** | **Third Moment Skewness** | **Fourth Moment Kurtosis** |
| **ASD1 *vs*. CT1a** | 1.0774 x10^-4^ | 1.0774 x10^-4^ | 1.0774 x10^-4^ | 0.8916 | 0.2129 |
| **ASD1 *vs*. CT1b** | 1.0774 x10^-4^ | 4.3094 x10^-4^ | 1.0774 x10^-4^ | 0.1025 | 0.4936 |
| **ASD1 *vs*. CT2** | 5.343 x10^-6^ | 2.529 x10^-5^ | 5.343 x10^-6^ | 0.0945 | 0.0043 |
| **ASD1 *vs*. ASD Parents** | 3.294 x10^-6^ | 0.0033 | 3.294 x10^-6^ | 0.7115 | 0.8916 |
| **ASD1 *vs.* CT3** | 4.163 x10^-6^ | 6.128 x10^-6^ | 4.163 x10^-6^ | 0.3011 | 5.454 x10^-5^ |
| **ASD1 *vs.* Elderly** | 3.969 x10^-5^ | 0.1673 | 3.969 x10^-5^ | 0.4824 | 0.9671 |
|  |  |  |  |  |  |
| **ASD2 *vs*. CT1a** | 0.0018 | 4.0669 x10^-4^ | 0.0014 | 1 | 0.0999 |
| **ASD2 *vs*. CT1b** | 6.8459 x10^-4^ | 0.0190 | 6.8459 x10^-4^ | 0.1152 | 0.5518 |
| **ASD2 *vs*. CT2** | 8.046 x10^-7^ | 6.976 x10^-5^ | 1.126 x10^-6^ | 0.0424 | 0.0023 |
| **ASD2 *vs*. ASD Parents** | 4.563 x10^-7^ | 0.4553 | 5.349 x10^-7^ | 0.3686 | 0.4193 |
| **ASD2 *vs.* CT3** | 3.366 x10^-7^ | 2.735 x10^-6^ | 3.366 x10^-7^ | 0.2813 | 1.636 x10^-5^ |
| **ASD2 *vs.* Elderly** | 2.299 x10^-4^ | 0.0133 | 2.9448 x10^-4^ | 0.3408 | 0.9494 |

**Supplementary Table 8:** Summary statistics of the ranges of first, second, third and fourth estimated moments for the normalized peak velocity index for the PD participants across severity stages and their statistical comparisons to controls using the non-parametric rank sum Wilcoxon test.

| **PD per Symptoms Group**  **Median and Ranges** | **Noise to Signal** | **First Moment Mean** | **Second Moment Variance** | **Third Moment Skewness** | **Fourth Moment Kurtosis** |
| --- | --- | --- | --- | --- | --- |
| **PD1 Mild**  **Median**  **Min**  **Max** | 0.0019  0.0012 0.0056 | 0.7004  0.6546 0.7240 | 0.0014  0.0008 0.0041 | 0.2879  -0.2117 0.6769 | 3.3029 2.3368 4.3287 |
| **PD2 Severe**  **Median**  **Min**  **Max** | 9.554 x10^-4^  0.0005 0.0013 | 0.6249  0.6105 0.6471 | 6.002 x10^-4^  3.194 x10^-4^ 7.780 x10^-4^ | 0.1521  -0.3626 0.5827 | 3.0493  2.5445 7.6453 |
| **Controls per Age Group**  **P value Rank Sum Test** | **Noise to Signal** | **First Moment Mean** | **Second Moment Variance** | **Third Moment Skewness** | **Fourth Moment Kurtosis** |
| **PD1 *vs*. CT2**  **(severe)** | 2.365 x10^-6^ | 2.365 x10^-6^ | 2.365 x10^-6^ | 0.5393 | 0.0072 |
| **PD1 *vs*. ASD Parents** | 0.2771 | 0.0027 | 0.2171 | 0.9266 | 0.0801 |
| **PD1 *vs.* CT3** | 4.150 x10^-6^ | 5.071 x10^-6^ | 4.150 x10^-6^ | 0.1319 | 9.454 x10^-4^ |
| **PD1 *vs.* Elderly** | 0.0572 | 0.0961 | 0.0476 | 0.5261 | 0.2346 |
|  |  |  |  |  |  |
| **PD2 *vs*. CT2**  **(mild)** | 1.227 x10^-6^ | 0.5401 | 1.450 x10^-6^ | 0.7033 | 6.097 x10^-4^ |
| **PD2 *vs*. ASD Parents** | 2.672 x10^-4^ | 2.271 x10^-6^ | 1.249 x10^-4^ | 0.2203 | 0.1388 |
| **PD2 *vs.* CT3** | 5.068 x10^-5^ | 0.7595 | 3.715 x10^-5^ | 0.0351 | 2.559 x10^-4^ |
| **PD2 *vs.* Elderly** | 3.507 x10^-4^ | 2.087 x10^-4^ | 2.087 x10^-4^ | 0.7133 | 0.7133 |
|  |  |  |  |  |  |
| **PD1 *vs.* PD2** | 8.544 x10^-6^ | 5.658 x10^-6^ | 5.658 x10^-6^ | 0.1672 | 0.3238 |

## Supplementary Table 9: Summary statistics of the ranges of first, second, third and fourth estimated moments for the normalized peak velocity index for the SZ participants grouped by age and their statistical comparisons to controls using the non-parametric rank sum Wilcoxon test.

| **SZ per Age Group**  **Median and Ranges** | **Noise to Signal** | **First Moment Mean** | **Second Moment Variance** | **Third Moment Skewness** | **Fourth Moment Kurtosis** |
| --- | --- | --- | --- | --- | --- |
| **SZ1 22-30**  **Median**  **Min**  **Max** | 0.007  0.005  0.013 | 0.6381 0.6180 0.6577 | 0.005  0.002  0.007 | 0.6023  -0.1149 1.6207 | 5.2386 3.6226 8.1890 |
| **SZ2 32-40**  **Median**  **Min**  **Max** | 0.0014 0.0006 0.0024 | 0.6335 0.6132 0.6948 | 0.0083 0.0004 0.0017 | 0.7106  -0.0826 2.0448 | 3.8237 2.5896 10.4118 |
| **SZ3 46-57**  **Median**  **Min**  **Max** | 0.0015 0.0004 0.0042 | 0.6287 0.5870 0.6945 | 0.0092 0.0002 0.0027 | 0.0337  -0.2987 1.5069 | 2.8754 2.3293 10.6979 |
| **SZ per Age Group**  **P value Rank Sum Test** | **Noise to Signal** | **First Moment Mean** | **Second Moment Variance** | **Third Moment Skewness** | **Fourth Moment Kurtosis** |
| **SZ1 *vs*. CT2** | 0.0022 | 0.1021 | 0.0022 | 0.0330 | 0.3555 |
| **SZ1 *vs*. ASD Parents** | 0.0089 | 0.0108 | 0.0048 | 0.0960 | 0.0025 |
| **SZ1 *vs.* CT3** | 0.0502 | 0.1366 | 0.0417 | 0.8755 | 0.7540 |
| **SZ1 *vs.* Elderly** | 0.0101 | 0.0025 | 0.0025 | 0.1061 | 0.0051 |
|  |  |  |  |  |  |
| **SZ2 *vs*. CT2** | 0.0014 | 0.2269 | 0.0018 | 0.0330 | 0.99 |
| **SZ2 *vs*. ASD Parents** | 0.2345 | 0.0832 | 0.2345 | 0.0528 | 0.0528 |
| **SZ2 *vs.* CT3** | 0.0061 | 0.3471 | 0.0061 | 0.6383 | 0.8142 |
| **SZ2 *vs.* Elderly** | 0.2677 | 0.0480 | 0.2677 | 0.0732 | 0.1490 |
|  |  |  |  |  |  |
| **SZ3 *vs*. CT2** | 1.523 x10^-4^ | 0.3223 | 1.809 x10^-4^ | 0.7632 | 0.1113 |
| **SZ3 *vs*. ASD Parents** | 0.8851 | 0.0197 | 0.7884 | 0.5773 | 0.0945 |
| **SZ3 *vs.* CT3** | 0.0035 | 0.4239 | 0.0035 | 0.3007 | 0.0343 |
| **SZ3 *vs.* Elderly** | 0.7914 | 0.0059 | 0.7242 | 0.9298 | 0.5962 |
